# Efficient Killing of Multidrug‐Resistant Internalized Bacteria by AIEgens In Vivo

**DOI:** 10.1002/advs.202001750

**Published:** 2021-03-02

**Authors:** Ying Li, Fei Liu, Jiangjiang Zhang, Xiaoye Liu, Peihong Xiao, Haotian Bai, Shang Chen, Dong Wang, Simon H. P. Sung, Ryan T. K. Kwok, Jianzhong Shen, Kui Zhu, Ben Zhong Tang

**Affiliations:** ^1^ Center for AIE Research College of Materials Science and Engineering Shenzhen University Shenzhen 518061 China; ^2^ National Center for Veterinary Drug Safety Evaluation College of Veterinary Medicine China Agricultural University No. 2 Yuanmingyuan West Rd Beijing 100193 China; ^3^ Department of Biomedical Engineering Southern University of Science and Technology No. 1088 Xueyuan Rd, Nanshan District Shenzhen 518055 China; ^4^ Department of Chemistry Hong Kong Branch of Chinese National Engineering Research Center for Tissue Restoration and Reconstruction Institute for Advanced Study Division of Life Science The Hong Kong University of Science and Technology Clear Water Bay Kowloon Hong Kong China

**Keywords:** aggregation‐induced emission luminogen, antibiotic, autophagy, internalized bacteria, MRSA

## Abstract

Bacteria infected cells acting as “Trojan horses” not only protect bacteria from antibiotic therapies and immune clearance, but also increase the dissemination of pathogens from the initial sites of infection. Antibiotics are hard and insufficient to treat such hidden internalized bacteria, especially multidrug‐resistant (MDR) bacteria. Herein, aggregation‐induced emission luminogens (AIEgens) such as *N*,*N*‐diphenyl‐4‐(7‐(pyridin‐4‐yl) benzo [*c*] [1,2,5] thiadiazol‐4‐yl) aniline functionalized with 1‐bromoethane (TBP‐1) and (3‐bromopropyl) trimethylammonium bromide (TBP‐2) (TBPs) show potent broad‐spectrum bactericidal activity against both extracellular and internalized Gram‐positive pathogens. TBPs trigger reactive oxygen species (ROS)‐mediated membrane damage to kill bacteria, regardless of light irradiation. TBPs effectively kill bacteria without the development of resistance. Additionally, such AIEgens activate mitochondria dependent autophagy to eliminate internalized bacteria in host cells. Compared to the routinely used vancomycin in clinic, TBPs demonstrate comparable efficacy against methicillin‐resistant *Staphylococcus aureus* (MRSA) in vivo. The studies suggest that AIEgens are promising new agents for the treatment of MDR bacteria associated infections.

## Graphical abstract

Aggregation‐induced emission luminogens (AIEgens) offer great potentials to track and dissect their modes of action in bacteria and pharmacokinetics processes in hosts. AIEgens serve as a novel class of antibiotics by triggering reactive oxygen species (ROS)‐mediated membrane damage and by activating mitochondria dependent autophagy to kill bacteria, which are promising adjuvants to boost mammalian cells for bacterial clearance and treatment of MRSA associated peritonitis.

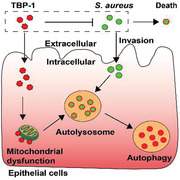

## 1 Introduction

**Scheme 1:**
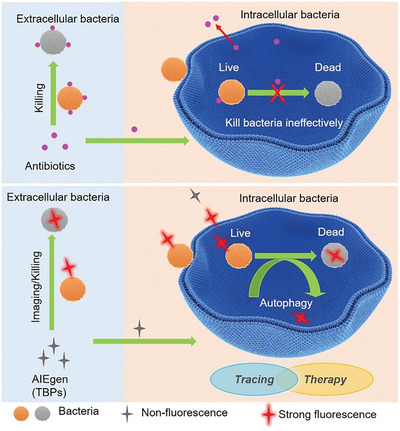
Antibiotics and AIEgen against extracellular bacteria and intracellular bacteria.

Pathogenic bacteria cause severe illness and significant mortality globally. The discovery of penicillin in 1928 and its subsequent introduction to clinic is a cornerstone of modern medical system. Since then, numerous classes of antibiotics have saved countless individuals from bacterial infections. However, such antibacterial abilities have been dramatically impaired due to the emergence and dissemination of antibiotic resistance.^[^ ^1^ ^]^ Infections associated with multidrug‐resistant (MDR) bacteria, such as methicillin‐resistant *Staphylococcus aureus* (MRSA) and vancomycin‐resistant *Enterococcus* (VRE), are increasing to overwhelm nosocomial treatments worldwide, leading to high mortalities.^[^ ^2^, ^3^, ^4^ ^]^ The Centers for Disease Control and Prevention (CDC) estimate that more than two million people suffer from antibiotic resistant infections and at least 23 000 people die each year in the United States alone.^[^ ^5^ ^]^ The rising rates of MDR bacterial pathogens pose a serious threat to public health.^[^ ^6^ ^]^ Additionally, infections with MDR bacteria are notorious to treat due to their invasion and survival in mammalian cells, exhibiting similar behaviors to typical intracellular bacteria. For example, *Staphylococcus aureus* (*S. aureus*) and *Klebsiella pneumoniae* are able to survive inside mammalian cells.^[^ ^7^, ^8^ ^]^ Bacteria infected cells act as “Trojan horses”, which not only protect bacteria from antibiotic treatments and immune clearance, but also may increase the dissemination of bacteria from the initial infection sites. Conventional antibiotics are difficult to kill such intracellular bacteria (**Scheme** **1**). Therefore, it is urgent to develop alternative strategies and antibiotics, for the better treatment of such MDR bacterial pathogens.

Antibacterial mechanisms of clinically available antibiotics are largely ascribed to targeting the imperative macromolecular biosynthetic processes, including cell‐wall assembly, DNA replication and transcription, and protein biosynthesis.^[^ ^9^ ^]^ The misuse and abuse of antibiotics have facilitated bacteria to evolve resistance against almost all existing antibiotics.^[^ ^10^ ^]^ The majority of antibiotics routinely used such as *β*‐lactam antibiotics, tetracyclines, and aminoglycosides are natural products and their derivatives. Unlike such natural compounds, synthetic chemicals have the potential to circumvent the coevolved resistance to target bacteria with distinctive modes of action.^[^ ^10^ ^]^ Inspired by the paradigms of sulfonamide and quinolone antibiotics, there are great prospects to mine the unexplored source of abundant synthetic chemicals for antibacterial purposes.

Although synthetic chemicals carrying unique scaffolds always endow them with the potential against MDR bacteria, it suffers from the lack of efficient methodologies to fish the leads, due to the low throughput of common screening assays, e.g., Waksman's platform. Multifunctional chemicals with antibacterial capabilities are potent candidates to advance antibiotic discovery. The phototherapy has been demonstrated as a promising alternative to treat MDR bacteria.^[^ ^11^ ^]^ Recently, aggregation induced emission luminogens (AIEgens), which are weak fluorescent or nonfluorescent in solution but displaying strong fluorescence by accumulating in the cytoplasmic membrane, have made great progresses in biosensing, imaging and antibacterial purposes.^[^ ^12^, ^13^, ^14^, ^15^ ^]^ Membrane targeting antibacterial agents always increase bacterial metabolic burden with high fitness cost for the development of resistance.^[^ ^16^ ^]^ Therefore, we hypothesized that the versatile AIEgens are promising leading compounds for screening membrane‐targeting antimicrobial agents.

Our previous reports have shown that different AIEgen families are phenomenally active against bacteria,^[^ ^17^, ^18^ ^]^ whereas the underlying mechanisms are still unclear. In the present study, we chose *N*,*N*‐diphenyl‐4‐(7‐(pyridin‐4‐yl) benzo [*c*] [1,2,5] thiadiazol‐4‐yl) aniline (TBP) functionalized with 1‐bromoethane (TBP‐1) and (3‐bromopropyl) trimethylammonium bromide (TBP‐2), as modeling molecules to explore their abilities against both extracellular and internalized MDR bacteria. TBPs showed high efficacy against diverse Gram‐positive bacteria through membrane disruption without the development of resistance after repetitive passages for 30 days. Additionally, TBPs eliminated the internalized pathogens via enhancing autophagy in vitro (Scheme 1) and in an animal model. These findings shed light on the development of next generation of AIEgens to combat MDR bacteria‐associated infections.

## 2 Results

### 2.1 TBPs Effectively Kill Multidrug‐Resistant Bacteria

**Figure 1:**
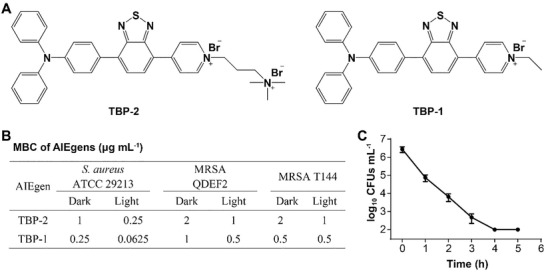
Chemical structures and bactericidal properties of TBP‐2 and TBP‐1. A) Chemical structure of TBP‐2 and TBP‐1. B) Bactericidal activities of TBP‐2 and TBP‐1 with and without light irradiation (*n* = 3), bacterial growth in MHB medium. MBC is the minimal concentration at which less than five colonies grow after the subculture on the agar plate post MICs tests. C) Bactericidal activity of *S. aureus* treated with TBP‐1 (10 × MICs, 2.5 µg mL^−1^) as a function of time in darkness (*n* = 3).

Previous reports indicate that quaternary ammonium compounds can effectively kill bacteria whereas are not easy to develop resistance.^[^ ^19^ ^]^ TBP‐2 (**Figure** **1**A) exhibited antibiotic activities against both antibiotic‐susceptible and MDR Gram‐positive bacteria including diverse MRSA and VRE isolates, with the minimal inhibition concentrations (MICs) ranging from 0.25 to 2 µg mL^−1^ in darkness (Table S1, Supporting Information). However, we found 4‐fold decrease of the MICs of TBP‐1 (Figure 1A) with the MICs ranging from 0.0625 to 0.5 µg mL^−1^ in darkness (Table S1, Supporting Information). Interestingly, the MICs of such chemicals decreased 2‐ to 4‐fold under 4 mW cm^−2^ light irradiation as well (Table S1, Supporting Information). Given the unmet and urgent need for treating MRSA‐associated infections in clinic,^[^ ^20^ ^]^ we chose either *S. aureus* ATCC 29213 or MRSA for the following mechanistic studies. Bactericidal activities of TBP‐2 and TBP‐1 were first tested by the minimal bactericidal concentration (MBC) assay.^[^ ^21^ ^]^ The values of MBC were the same or only 2‐fold higher than their corresponding MICs (Figure 1B), suggesting that such chemicals have bactericidal activity. As expected, TBP‐1 (10 × MICs, 2.5 µg mL^−1^) killed almost all *S. aureus* ATCC 29213 within 1 h in darkness based on time‐kill analysis (Figure 1C), and TBP‐2 (10 × MICs, 5.0 µg mL^−1^) killed almost all *S. aureus* within 3 h (Figure S1, Supporting Information). The positively charged TBPs have been proposed to target bacteria via electrostatic interactions.^[^ ^19^ ^]^ The zeta potentials of TBP‐1 and TBP‐2 are 12.37 ± 1.36 and 10.53 ± 0.41, respectively. TBP‐2 has lower zeta potential (Figure S2, Supporting Information) and smaller size than TBP‐1 (Figure S3, Supporting Information). TBP‐2 needs to overcome larger energy barrier to go through cell membrane and have lower permeability coefficient than TBP‐1, because the additional positive charge of TBP‐2 may further reduce its efficiency across the cell membrane.^[^ ^22^ ^]^ In addition, the hydrophobicity indicated by the calculated log *P* value (*n*‐octanol/water partition coefficient, *C* log *P*), can also influence the behavior of the TBP molecules. The *C* log *P* of TBP‐2 and TBP‐1 are −0.7 and 3.5, respectively, consistent with the fact that high concentration of TBP‐2 is needed to kill bacteria.^[^ ^13^, ^23^ ^]^ Thus, TBP‐1 is a promising candidate to rapidly kill bacteria and for the following mechanistic study.

### 2.2 TBPs Trigger Membrane Damage in Gram‐Positive Bacteria

**Figure 2:**
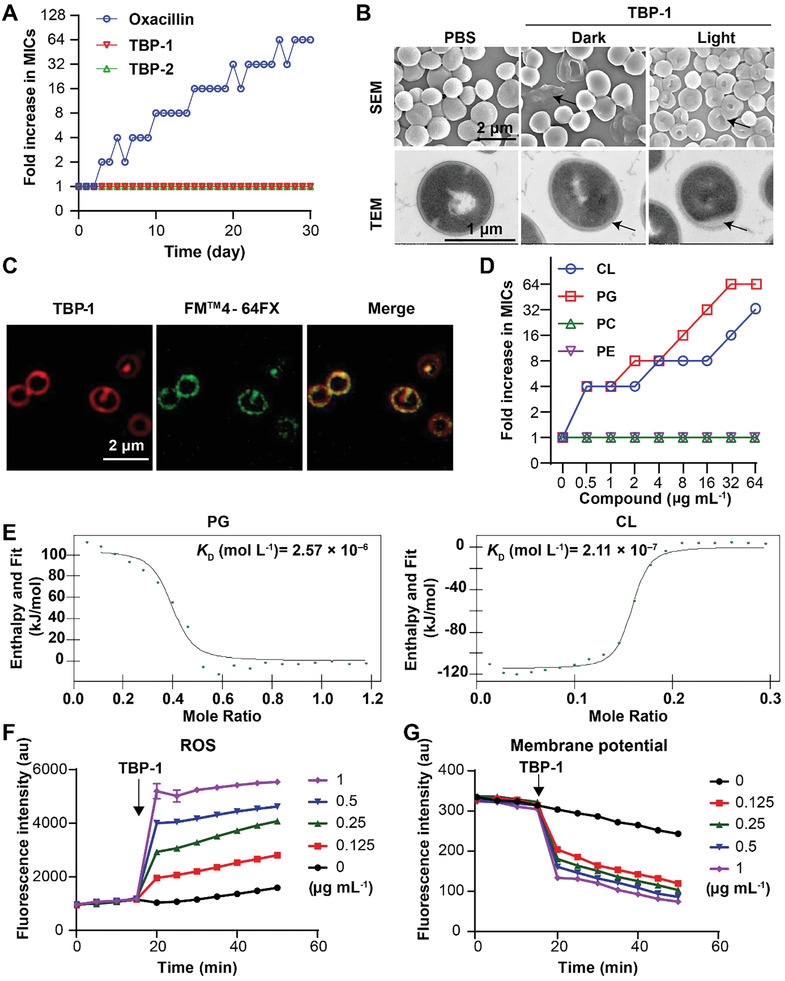
TBP‐1 killed *S. aureus* by targeting bacterial membrane. A) Fold increase of MICs after repetitive treatments with TBP‐1, TBP‐2, or oxacillin for 30 days in the darkness. Oxacillin served as a positive control. B) Morphology of *S. aureus* ATCC 29213 incubation with TBP‐1 (dark, 0.25 µg mL^−1^; light, 0.0625 µg mL^−1^) with and without white light irradiation (4 mW cm^−2^) were imaged by FE‐SEM and TEM (dark, 2.5 µg mL^−1^; light, 0.625 µg mL^−1^), bacteria without treatment were set as controls. The black arrows represent bacteria with collapsed and lysed structure. C) Super‐resolution SIM images of *S. aureus* incubation with TBP‐1 (1 µg mL^−1^, red) and FM 4–64FX (5 µg mL^−1^, green) for 5 min. TBP‐1: Ex = 488 nm, Em = 600–700 nm; FM 4–64FX: Ex = 640 nm, Em = 650–700 nm. D) Increased MICs of TBP‐1 against *S. aureus* in the presence of CL, PG, PC, and PE, ranging from 0 to 64 µg mL^−1^. E) ITC analysis of the interaction between TBP‐1 and PG/CL; 2 × 10^−3^ mol L^−1^ POPG was dropped into 0.5 mmol L^−1^ TBP‐1 in HEPES buffer at 25 °C. Thermodynamic parameters were calculated, including the equilibrium dissociation constant (*K*_D_ = 2.57 × 10^−6^ mol L^−1^), molar binding enthalpy (Δ*H* = 21.68 kJ mol L^−1^) and molar binding entropy (Δ*S* = 179.7 J mol^−1^ K^−1^). 0.5 mmol L^−1^ CL was dropped into 0.5 mmol L^−1^ TBP‐1 in 10% alcohol at 25 °C. Thermodynamic parameters were calculated, including the equilibrium dissociation constant (*K*_D_ = 2.11 × 10^−7^ mol L^−1^), molar binding enthalpy (Δ*H* = −91.73 kJ mol^−1^), and molar binding entropy (Δ*S* = −179.8 J mol^−1^ K^−1^). F) Plot of the internalized ROS fluorescence intensity of *S. aureus* after incubation with TBP‐1 (0.125, 0.25, 0.5, and 1 µg mL^−1^) as a function of time. G) Plot of membrane potential (Δ*Ψ*_m_) fluorescence intensity of *S. aureus* after incubation with TBP‐1 (0, 0.125, 0.25, 0.5, and 1 µg mL^−1^) with the time increased.

As TBPs effectively kill bacteria, we next explored the resistance to TBPs, and found that the development of resistance to TBPs was not observed after a serial passage of *S. aureus* in the presence of subinhibitory levels of TBPs over 30 days in the darkness (**Figure** **2**A). To further understand the bactericidal role of TBPs, we employed the field emission scanning electron microscopy (FE‐SEM) and transmission electron microscope (TEM) to visualize the morphological changes of bacteria. *S. aureus* ATCC 29213 and MRSA T144 exhibited wrinkling, collapsed and lysed structures after the treatment with TBPs in the darkness, while light irradiation aggravated the morphological damage to bacteria (Figure 2B; Figures S4–S6, Supporting Information). We observed that bacteria could be immediately stained by TBPs (Figure S7, Supporting Information), which can be utilized to get better understanding of AIEgen‐mediated antibacterial activity. Subsequently, we used TBP‐1 as a model to colabel bacterial membrane and cell wall with commercial dyes (FM 4‐64FX and WGA Alexa Fluor 640).^[^ ^24^ ^]^ It showed that TBP‐1 mainly bound to bacterial membrane (Figure 2C; Figure S8, Supporting Information). Meanwhile, we found that the entire bacterial body were stained by TBPs, suggesting that TBPs efficiently accumulated in *S. aureus* (Figures S9 and S10, Supporting Information). Lastly, TBPs disrupted the membrane permeability of *S. aureus* after incubation for 10 min, based on propidium iodide (PI) analysis (Figure S11, Supporting Information). These results indicate that TBPs mainly cause membrane damage to *S. aureus* to induce bacterial death.

To gain an insight into the potential target of TBPs, we compared the antibacterial activity of TBPs with three major membrane phospholipids of bacteria,^[^ ^25^ ^]^ including phosphatidylethanolamine (PE), phosphatidylglycerol (PG), and cardiolipin (CL) (Table S2, Supporting Information). Additionally, we assessed the antibacterial activity of TBPs with the components of bacterial wall lipoteichoic acid (LTA) and peptidoglycan (wall teichoic acids, WTA).^[^ ^26^ ^]^ We found that PG and CL specially abolished the activity of TBP‐1 and TBP‐2 in a dose‐dependent manner (Figure 2D; Figure S12, Supporting Information). These results revealed that TBPs showed high affinity to CL and PG. Therefore, isothermal titration calorimetry (ITC) assays were performed to further evaluate the interaction using TBP‐1, PG/CL. The result showed that the equilibrium dissociation constant (*K*_D_) was 2.57 × 10^−6^ mol L^−1^ with the stoichiometric ratio of PG to TBP‐1 at 0.42, *K*_D_ was 2.1 × 10^−7^ mol L^−1^ with the stoichiometric ratio of CL to TBP‐1 at 0.151 (Figure 2E; Figure S13, Supporting Information). Thus, we deduce that PG and CL in *S. aureus* may be the potential targets of TBPs.

Given that many bactericidal antibiotics display ROS dependent activity against bacteria,^[^ ^9^, ^27^ ^]^ we observed that TBPs leading to the accumulation of ROS in a dose dependent manner (Figure 2F; Figure S14, Supporting Information). Exogenous addition of *N*‐acetyl cysteine (NAC, a ROS scavenger) significantly decreased the ROS level and enhanced the survival of *S. aureus* in the presence and absence of white light irradiation (Figure S15, Supporting Information). In addition, we confirmed that TBPs as a photosensitizer generates ROS under white light irradiation (Figure S16, Supporting Information), consistent with previous reports.^[^ ^28^ ^]^ Taken together, these findings indicate that TBPs probably bind to the phospholipid PG and CL, resulting in membrane damage by inducing ROS accumulation to kill bacteria.

### 2.3 TBPs Kill Internalized *S. aureus* through Enhancing Autophagy

**Figure 3:**
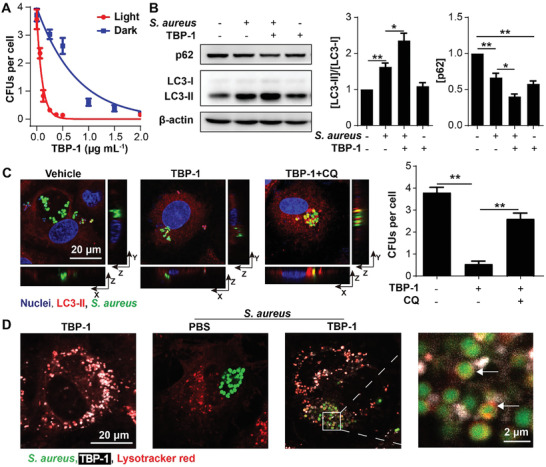
TBP‐1 killed the internalized *S. aureus* through enhancing autophagy of IEC‐6 cells. A) Bactericidal activity of internalized *S. aureus* treated with different concentrations of TBP‐1 with and without light irradiation. B) Western blot analysis of Microtubule Light Chain 3 (LC3) and p62 expression in IEC‐6 cells infected with or without *S. aureus* in the presence and absence of TBP‐1 (1 µg mL^−1^). All proteins were normalized to the level of *β*‐actin. C) Fluorescent images and CFUs of internalized *S. aureus* after IEC‐6 cells incubated with *S. aureus, S. aureus* + TBP‐1 and a lysosome inhibitor chloroquine (CQ) + *S. aureus* + TBP‐1. IEC‐6 cells were stained with LC3 (Ex = 552 nm, Em = 565 nm) and DAPI (Ex = 405 nm, Em = 454 nm). D) Fluorescent images of IEC‐6 cells treated with TBP‐1, *S. aureus* (green) + PBS, *S. aureus* + TBP‐1, and then stained with lysotracker red (Ex = 552 nm, Em = 590 nm). Magnification images of the outlined area are shown on the right, the white arrows indicate the overlay of lysotracker, TBP‐1 and *S. aureus*. ns not significantly, *P < 0.05, **P < 0.001.

In view of the promising bactericidal activity of TBPs, we tested their antibacterial ability against internalized bacteria. Compared to TBP‐2, TBP‐1 exhibited low values of MIC and MBC against MRSA isolates under light irradiation (Tables S3 and S4, Supporting Information). Also, TBP‐1 had low toxicity with high therapeutic index in the darkness (Table S5, Supporting Information). To test whether TBP‐1 can target internalized bacteria, we infected IEC‐6 cells with *S. aureus* ATCC 29 213 at multiplicity of infection (MOI) of 1. The increased infection rate of internalized bacteria was in a time dependent manner (Figure S17 A‐B Supporting Information). Nevertheless, the number of internalized bacteria sharply diminished under the treatment of TBP‐1, which was further aggravated under light irradiation (**Figure** **3**A). Given that autophagy plays an essential role in enabling eukaryotic organisms to clear bacterial invasion,^[^ ^29^ ^]^ we subsequently determined whether autophagy contributes to TBPs‐mediated killing of internalized bacteria. We first evaluated the expression levels of autophagy‐related proteins,^[^ ^30^ ^]^ including LC3‐II, LC3‐I, and p62 in IEC‐6 cells infected with *S. aureus*. We observed the increasing ratio of LC3‐II/LC3‐I and declining level of p62 during the infection time (Figure S18A,B, Supporting Information). Furthermore, we observed that the number of internalized *S. aureus* increased with the coculture of autophagy inhibitor chloroquine (CQ), while the agonist rapamycin (Rapa) reduced the number of internalized bacteria (Figure S18C,D, Supporting Information).^[^ ^31^ ^]^ These findings indicate the activation of autophagy in IEC‐6 cells infected with *S. aureus*, agreeing with previous reports that bacteria can persist in the cytoplasm of mammalian cells by hijacking autophagy.^[^ ^32^, ^33^ ^]^ In addition, *S. aureus* escaped the degradation by lysosome, indicating the inhibition of lysosomal acidification (Figure S18E, Supporting Information). Together, these results suggest that *S. aureus* survival and replicate in host cells through hijacking autophagy. To gain detailed understanding of TBP‐1 killing internalized *S. aureus*, we dissected the effect of TBP‐1 on the process of autophagy in IEC‐6 cells infected with *S. aureus*. TBP‐1 treatment boosted autophagy by increasing the ratio of LC3‐II/LC3‐I and reducing the level of p62 (Figure 3B; Figure S19, Supporting Information). To gain more insight, cytochalasin D was used to explore whether TBP‐1 affects phagocytosis. The results showed that TBP‐1 was not potentiated in the presence of cytochalasin D and no obvious effect on phagocytosis (Figure S20, Supporting Information). Compared to the treatment with CQ, we observed the decreased number of internalized bacteria in the presence of TBP‐1 (Figure 3C; Figure S21, Supporting Information). Furthermore, we found that the colocalization of TBP‐1 with lysosome and *S. aureus* in IEC‐6 cells (Figure 3D). It suggested that TBP‐1 might facilitate or reverse the function of lysosome to kill internalized *S. aureus*, based on Western blot analysis (Figure S22, Supporting Information). Altogether, these results suggest that TBPs eliminate the internalized *S. aureus* through enhancing the autophagy in mammalian cells.

### 2.4 ROS Plays a Key Role in TBP‐Induced Killing of Internalized *S. aureus*

**Figure 4:**
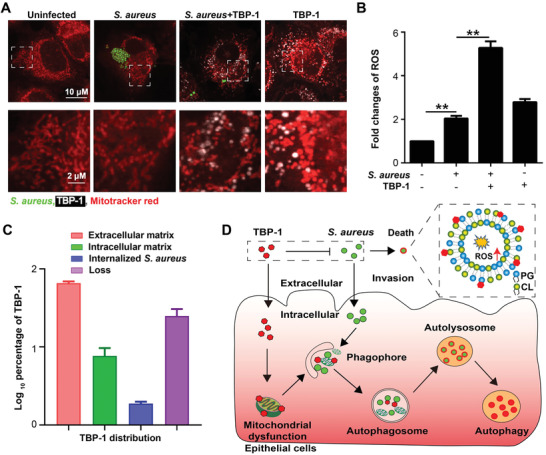
TBP‐1 enhanced autophagy through inducing mitochondrial dysfunction. A) Morphology and structure changes of mitochondria after IEC‐6 cells incubated with and without *S. aureus* (green), *S. aureus* + TBP‐1 and TBP‐1. The mitochondria stained by Mito‐tracker red (Ex = 552 nm, Em = 600 nm). The white rectangles indicate the magnified part of the bottom images. B) Fold changes of ROS in IEC‐6 cells infected with or without *S. aureus* in the presence and absence of TBP‐1 (**P < 0.001). C) Relative amount of TBP‐1 in medium, IEC‐6 cells, *S. aureus* and loss in in the process of experimental treatment. The quantified of TBP‐1 was measured by LC–MS/MS. D) Schematic illustration of the mechanism of TBP‐1 mediated antibacterial activity against either the extracellular *S. aureus* or internalized bacteria in host cells.

To further understand how TBP‐1 enhanced autophagy, we hypothesized that mitochondrial perturbation contributes to the clearance of invaded bacteria. We found that the internalized *S. aureus* slightly caused morphological damage to mitochondria, whereas the addition of TBP‐1 dramatically aggravated such damage (**Figure** **4**A). Damaged mitochondria resulted in the accumulation of ROS (Figure 4B). Similarly, the co‐incubation of TBP‐2 increased the ROS levels as well (Figure S23, Supporting Information). To clarify the underlying mechanisms, we further investigated the membrane potential (Δ*Ψ*_m_) of mitochondria in IEC‐6 cells based on the cyanine dye 5,5″,6,6″‐tetrachloro‐1,1″,3,3″ tetraethylbenzimidazolyl‐carbocyanine iodide (JC‐1) staining assay.^[^ ^34^ ^]^ We observed the increase of green fluorescent JC‐1 monomers (Figure S24A, Supporting Information) with high ratio of green/red fluorescence under TBP‐1 treatment (Figure S24B, Supporting Information). These results suggest that TBP‐1 paralyzed the homeostasis of mitochondria, which may promote ROS production. Exogenous addition of ROS scavenger NAC significantly blocked the clearance of internalized *S. aureus* in the presence of TBP‐1 (Figure S25, Supporting Information). It indicates that ROS plays a critical role in TBP‐1‐mediated clearance of *S. aureus*. Furthermore, we quantified TBP‐1 in the cytosol of IEC‐6 cells infected with *S. aureus* based on liquid chromatography‐tandem mass (LC–MS/MS) analysis (Figure S26 and Table S6, Supporting Information). The amount of TBP‐1 in cells consisted about 6% of the total (Figure 4C), which may be easily enriched to high levels in the limited space to clear bacteria in mammalian cells. Collectively, our results indicate that TBPs mediated bactericidal activity against *S. aureus* is probably through the direct disruption of bacterial membrane and indirect potentiation of autophagy to eliminate invasive bacteria (Figure 4D).

### 2.5 TBPs Protect Mice from MRSA Associated Infection

**Figure 5:**
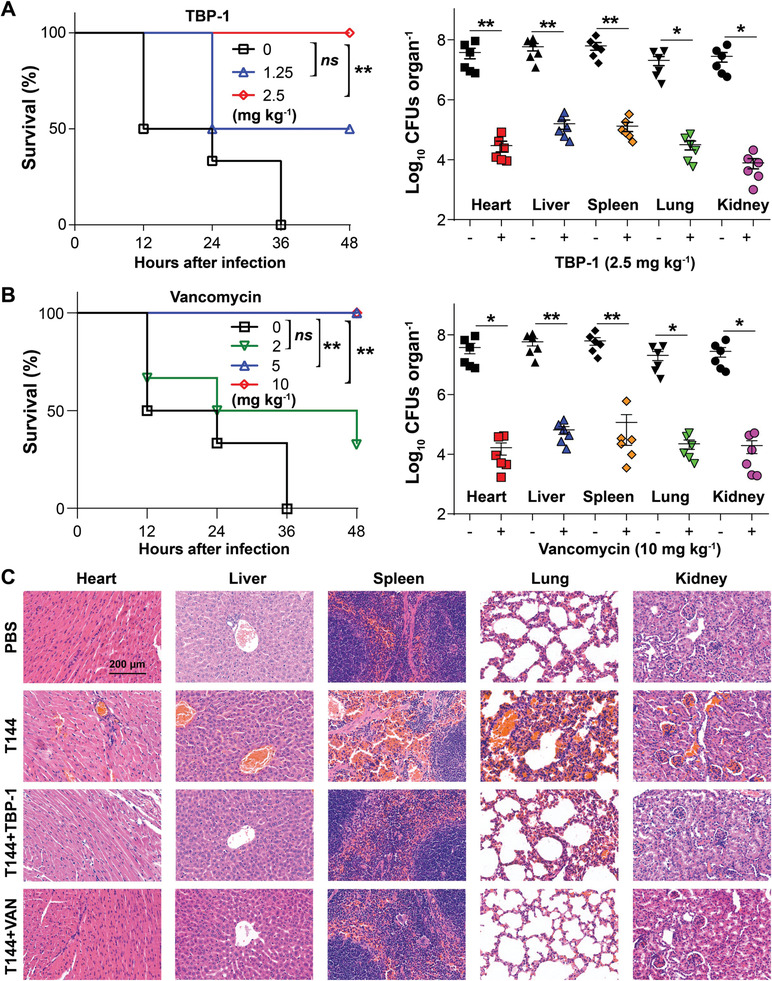
Efficacy of TBP‐1 and vancomycin in the mouse peritonitis model. Survival rate and bacteria survival in different organs of mice after treatment with A) TBP‐1 and B) Vancomycin in septicemia protection model using MRSA T144 (*n* = 5, ns not significantly; *P < 0.05; **P < 0.001). C) Histological hemotoxylin and eosin (HE) staining images of heart, liver, spleen, lung, and kidney tissue sections at day 2 after different treatments indicated.

Given the promising antibacterial activity of TBPs in vitro, we evaluated their therapeutic effects on a mouse peritonitis model. Mice were injected intraperitoneally with MRSA T144 with single dose that lead to 90% lethality.^[^ ^20^ ^]^ TBPs were introduced intraperitoneally at 1h postinfection. The infected mice without treatment all died within 48 h, while the mice survived under the treatment of either TBP‐1 (**Figure** **5**A) or vancomycin (5 mg kg^−1^) (Figure 5B). Meanwhile, bacterial counts in different organs decreased significantly in the presence of 2.5 mg kg^−1^ TBP‐1 (Figure 5A), which showed comparable numbers in the organs treated with 10 mg kg^−1^ vancomycin (Figure 5B). Similarly, all mice survived in the presence of 5 mg kg^−1^ TBP‐2, with decreased bacterial counts in the organs (Figure S27A,B, Supporting Information). Lastly, typical pathologic changes of the organs were alleviated in the treated groups as well (Figure 5C). However, the organs showed hemorrhage and congestion after MRSA T144 infection, especially the spleen and lung (Figure 5C; Figure S27C, Supporting Information).

**Figure 6:**
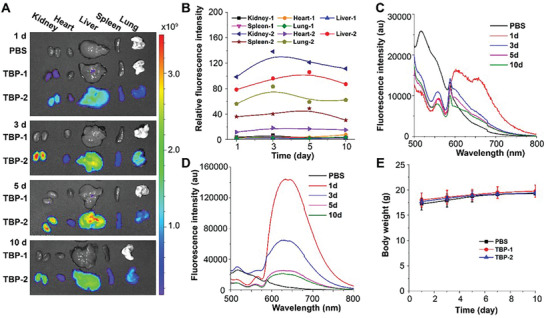
Accumulation and clearance of TBPs in organs. A) The ex vivo images of the major organs of mice at 1, 3, 5, 10 days postinjection with PBS, TBP‐1 (2.5 mg kg^−1^), and TBP‐2 (5 mg kg^−1^), respectively. B) The average fluorescence intensity of TBPs in different organs. The fluorescence intensity of C) TBP‐1 and D) TBP‐2 in blood at different time postinjection (Ex = 488 nm, Em = 500–800 nm). E) Body weight changes of the mice (*n* = 3).

To get better understanding of the pharmacokinetic behavior of TBPs, we analyzed the distribution of TBPs in mice based on their intrinsic fluorescent properties. Compared to the wide and persistent distribution of TBP‐2, there was weak fluorescence signal in the kidney of mice treated with TBP‐1 for 1 day (**Figure** **6**A–D). The body weight of mice showed no difference under the treatment of TBPs (Figure 6E), suggesting their low systematic toxicity. Last, we quantified the metabolism of TBPs in vitro by the ultrahigh performance liquid chromatography‐quadrupole time‐of‐flight mass spectrometry (UHPLC/Q‐TOF‐MS) technique using TBP‐1 as a model. Most TBP‐1 was in the prototype form, and only a small fraction was oxidized (Figure S28, Supporting Information). Altogether, these results demonstrated that TBPs particular TBP‐1 have promising pharmacokinetic properties, which are potent agents for the treatment of MRSA associated infections in vivo.

## 3 Discussion

Antibiotics that target bacterial membrane always increase metabolic burden, therefore it is not easy to develop antibiotic resistance due to high fitness cost.^[^ ^35^ ^]^ However, membrane‐targeting antibiotics have been rarely exploited. Similar to the synthetic antibacterial compounds derived from sulfonamides and quinolones,^[^ ^36^ ^]^ the versatile skeletons of AIEgens provide tremendous sources for developing leading compounds to circumvent antibiotic resistance with distinctive mechanisms. Notably, the intrinsic fluorescent property of AIEgens offers great potentials to track and dissect their modes of action in bacteria and pharmacokinetics processes in hosts. Figure 4D shows the potential mechanisms of AIEgens particularly for TBP‐1 mediated antibacterial activity against *S. aureus* in the presence and absence of host cells. We get insightful understandings of TBP‐mediated antibacterial activities, which directly disrupts bacterial membrane and indirectly potentiates host cells to clean invasive bacteria.

The cell wall of Gram‐positive bacteria is composed of cross‐linked peptidoglycans and anionic teichoic acids. Cationic TBPs may bind to the negatively charged teichoic acids, facilitating the insertion into the porous structure of cell wall to further disrupt membrane integrity to cause bacterial death. Nevertheless, such electrostatic interaction can be impaired in Gram‐negative bacteria, due to the presence of thick and highly hydrophobic layer of lipopolysaccharide. This observation agrees with our previous findings that adequate modification of certain groups (hydrophilic amines,^[^ ^37^ ^]^ naphthalimide triazole,^[^ ^15^ ^]^ antimicrobial peptide^[^ ^38^ ^]^) in the AIEgen skeletons promote antibacterial activities against Gram‐negative pathogens. Altogether, it suggests that the pyridine ring of TBP is a crucial position to introduce functional groups to extend the antibacterial spectrum.

Unlike antibiotics that directly target pathogens, host‐directed antibacterial therapy is an alternative approach to potentiate hosts for further elimination of invasive bacteria.^[^ ^39^ ^]^ Notably, autophagy, as a ubiquitous response in host cells, plays a pivotal role in the defense against the invasion of bacterial pathogens.^[^ ^40^ ^]^ In this study, TBPs not only trigger ROS mediated membrane damage to kill bacteria, but also activate mitochondria‐dependent autophagy process to eliminate internalized bacteria in host cells. In this scenario, versatile TBPs serve as a novel class of antibiotic adjuvants to boost bacterial clearance in mammalian cells. Future studies are urgently required to demonstrate whether and how such antibiotic adjuvants work in vivo under pathophysiological conditions, guiding the optimization of the structure–activity relationship of AIEgen molecules for tracing and killing MDR bacterial pathogens.

## 4 Experimental Section

##### Materials

All bacterial strains used in this study are listed in Table S1 (Supporting Information). Bacteria were grown in brain heart infusion (BHI) broth (Land Bridge Technology) or on BHI agar plates at 37 °C. IEC‐6, A549, HUVEC, and 3T3 cells (Table S7, Supporting Information) were grown in Dulbecco's modified Eagle medium (DMEM, Gibco) supplemented with 1% (w/v) heat inactivated fetal bovine serum (FBS, Invitrogen), 1% (w/v) penicillin–streptomycin, and 1% (w/v) sodium pyruvate (Sigma‐Aldrich). Vancomycin was obtained from China Institute of Veterinary Drug Control.

##### Antimicrobial Assay

MICs of TBPs were determined by broth microdilution according to the Clinical and Laboratory Standards Institute (CLSI) 2018 guideline.^[^ ^41^ ^]^ After 16–20 h incubation at 37 °C, MIC values were defined as the lowest concentrations of antibiotics with no visible growth of bacteria. The bacteria colonies grown on the agar plate were counted and the MBC (less than five colonies) of each sample were obtained after MICs tests.^[^ ^42^ ^]^

Time‐dependent killing kinetic profiles were performed on the basis of a previous study.^[^ ^21^ ^]^ Overnight culture of *S. aureus* ATCC 29213 was 10 000 folds diluted in 1 mL MHB medium and incubated at 37 °C with shaking at 200 rpm. Bacterial populations at exponential phase (4 h) were challenged with TBPs at concentrations corresponding to the 10‐fold MICs (2.5 µg mL^−1^ TBP‐1, 5 µg mL^−1^ TBP‐2). After incubation at 37 °C with shaking at 200 rpm, serially diluted suspensions at different time points were plated on *S. aureus* chromogenic agar plates (CHROM agar) and incubated at 37 °C for overnight. Colonies were counted and colony forming unit (CFU mL^−1^) were calculated. Experiments were performed with three replicates.

##### Resistance Development Studies

For resistance, the overnight culture of *S. aureus* ATCC 29213 was diluted 1:100 in 1 mL MHB, then added 0.25, 0.5, 1, 2 and 4× MIC concentration of TBP‐1 and TBP‐2 in the darkness. Furthermore, oxacillin (MICs 0.25 µg mL^−1^) served as a positive control. The MIC was determined by broth microdilution after 24 h. With the change of MICs, the added drug concentration was increased in turn to ensure sufficient drug selection pressure.

##### Antibacterial Activity of the Mixtures of TBPs and Lipids

To assess the effects of various lipids on the antibacterial activity of TBPs, TBP was mixed with 2.5 × molar equivalent of PC (Sigma‐Aldrich, ≥ 99%), PG (Sigma‐Aldrich, ≥ 99%), PE (Sigma‐Aldrich, ≥ 99%), or CL (Sigma‐Aldrich, ≥ 99%). All lipids were dissolved in methanol. Mixtures were incubated at 37 °C for 30 min, then added to diffusion discs. After drying, the discs were placed on a tryptic soy agar (TSA) plate containing *S. aureus* ATCC 29213 as an indicator organism. After overnight incubation at 37 °C, the inhibition zones of the TBP‐lipid mixture were photographed and measured. In addition, the effect of various phospholipids (0–64 µg mL^−1^) on the antibacterial activity of TBPs in MHB medium using the chequerboard microdilution assay was further evaluated. Last, the turbidity of TBP‐lipid mixtures (100 µL) was measured, based on the absorbance at 600 nm in a flat‐bottomed, polystyrene, 96‐well plate (Corning).

##### ITC Assay

Calorimetric experiments were performed to evaluate the interaction between POPG/CL (Sigma‐Aldrich, ≥ 98%) and TBP‐1 by Affinity ITC (TA Instruments) at 25 °C. To determine the affinity between TBP‐1 and PG/CL, both 2 mmol L^−1^ of POPG and 0.5 mmol L^−1^ of TBP‐1 were dissolved in 4‐(2‐hydroxyethyl)‐1‐piperazine‐ethanesulfonic acid (HEPES, 20 × 10^−3^ m, pH 7.0); both 0.5 mmol L^−1^ of CL and 0.5 mmol L^−1^ of TBP‐1 were 10% alcohol at 25 °C. Sequential injections of PG/CL into the calorimetric cell filled with TBP‐1 were repeated 19 times with equilibration intervals of 200 s. The obtained data were processed using the software with the instrument to calculate the equilibrium dissociation constant (*K*_D_), stoichiometry (*n*), and changes of enthalpy (Δ*H*) and entropy (Δ*S*).^[^ ^43^ ^]^

##### ROS Detection

The levels of ROS were detected by ROS Assay Kit (Beyotime, China). The 2,7‐dichlorodi‐hydrofluorescein diacetate (DCFH‐DA) turned to 2,7‐dichlorofluorescein (DCF) (green fluorescence) represented ROS release (Ex = 488 nm, Em = 525 nm). The fluorescence intensity of ROS in *S. aureus* after incubation with TBP‐1 (0, 0.125, 0.25, 0.5, and 1 µg mL^−1^) or TBP‐2 (0, 0.25, 0.5, 1, and 2 µg mL^−1^) from 0 to 180 min was measured using a microplate reader (Tecan, Infinite 200 Pro Microplate Reader, Switzerland). The fluorescence intensity of ROS in IEC‐6 cells infected with or without *S. aureus* in the presence and absence of TBP‐1 (1 µg mL^−1^) or TBP‐2 (2 µg mL^−1^) was recorded accordingly.

##### Bacteria Membrane Potential Assay

Membrane permeability and dissipated membrane potential (Δ*Ψ*_m_) of *S. aureus* ATCC 29213 induced by TBP‐1 (0.125, 0.25, 0.5, 1 µg mL^−1^) were tested by the fluorescent dyes (10 nmol L^−1^) 3,3‐dipropylthiadicarbocyanine iodide (DiSC_3_ (5)) (Aladdin) according to a previous report.^[^ ^44^ ^]^ Experiments were performed with three replicates.

##### Membrane Permeability Test

Membrane permeability of *S. aureus* ATCC 29213 induced by TBPs was tested by the fluorescent dye (10 nmol L^−1^) propidium iodide (PI) (Aladdin) according to a previous report.^[^ ^45^ ^]^

##### Cell Viability Assay

IEC‐6, A549, HUVEC, and 3T3 cells were cultured in DMEM medium, which contained 1% FBS, 1% (w/v) penicillin–streptomycin and 1% (w/v) sodium pyruvate. Cells were seeded in 96‐well plates (0.5 × 10^4^ cells 200 µL^−1^) and incubated for 24 h. The medium was removed and TBPs were added at different concentrations and incubated in darkness for 30 min at 37 °C. The TBP‐treated cells were exposed to white light for 30 min, and the TBP‐treated cells in darkness were set as control. The cell medium was replaced by fresh medium and incubated for 24 h at 37 °C after irradiation. 100 µL of fresh medium containing 10 µL WST‐1 (Roche, Germany) solution was added into each well after the removal of the cell medium, and the cells were incubated at 37 °C for 1 h. The absorption of the samples was measured on a microplate reader (Tecan, infinite M200, Switzerland) at 450 nm. Cell viability (IC_50_) was determined by the concentrations of analytes of target resulting in 50% inhibition of cell growth.

##### Bacteria Culturing, Staining, and Imaging

*S. aureus* ATCC 29213 were cultured in the BHI broth medium at 37 °C with a shaking speed of 200 rpm for 3 h. Bacteria were harvested by centrifuging at 8000 rpm for 3 min and washed twice with phosphate buffered saline (PBS). Costaining: 1 mL of TBP‐1 (1 µg mL^−1^) or TBP‐2 (1 µg mL^−1^) solution in BHI broth medium containing 5 × 10^8^ CFU mL^−1^ of bacteria incubated at 37 °C with a shaking speed of 200 rpm for 5 min. To take fluorescence images, 2 µL of stained bacteria solution was transferred to a piece of glass slide and then covered by a coverslip. The images were collected using a stimulated emission of depletion (STED) microscopy (Leica SP8 STED 3X, Germany). TBP‐1 (1 µg mL^−1^) and FM4‐64FX (5 µg mL^−1^) containing 5 × 10^8^ CFU mL^−1^ of bacteria incubated at 37 °C with a shaking speed of 200 rpm for 5 min.TBP‐1 (1 µg mL^−1^) and WGA Alexa Fluor 640 (5 µg mL^−1^) containing 5 × 10^8^ CFU mL^−1^ of bacteria incubated at 37 °C with a shaking speed of 200 rpm for 10 min. To take fluorescence images, 2 µL of stained bacteria solution was transferred to a piece of glass slide and then covered by a coverslip. The images were collected using super‐resolution SIM microscopy (N‐SIM/N‐STORM010899, Nikon, Japan). 1 mL of TBP‐1 (2 µg mL^−1^) or TBP‐2 (2 µg mL^−1^) solution in BHI broth medium containing 5 × 10^8^ CFU mL^−1^ of bacteria incubated at 37 °C with a shaking speed of 200 rpm for 5 and 20 min; 1 mL of TBP‐1 (10 × MICs, 2.5 µg mL^−1^) and TBP‐2 (10 × MICs, 5 µg mL^−1^) for solution in BHI broth medium containing 5 × 10^8^ CFU mL^−1^of bacteria incubated at 37 °C with a shaking speed of 200 rpm for 10 min, and then incubated with (10 µmol L^−1^) propidium iodide (PI) for 10 min. To take fluorescence images, 2 µL of stained bacteria solution was transferred to a piece of glass slide and then covered by a coverslip. The images were collected using ultrahigh resolution confocal microscopy (ZEISS‐LSM880, Germany).

##### FE‐SEM Analysis

Bacterial cultures of *S. aureus* ATCC 29213 at mid‐log phase in BHI medium were collected and resuspended into PBS at cell density of ≈10^10^ CFU mL^−1^. After treatment of bacteria with TBP‐1 (dark, 0.25 µg mL^−1^; light, 0.0625 µg mL^−1^) and TBP‐2 (dark, 1 µg mL^−1^; light, 0. 25 µg mL^−1^) with or without light irradiation for 30 min, bacteria were collected and fixed in 2.5% glutaraldehyde solution. After fixation, samples were washed with 0.1 m PBS and dehydrated by 30%, 50%, 70%, 80%, 90%, 95%, and 100% (v/v, in water) ethanol for analysis by FE‐SEM (S4800, Hitachi, Japan).

##### TEM Analysis

5 × 10^8^ CFU mL^−1^ bacteria were dispersed in 1 mL of PBS solution in 1.5 mL microcentrifuge tube. The TBP‐treated bacteria solution was incubated at 37 °C with a shaking speed of 200 rpm for 2 h and then exposed to white light for 15 min. The TBP‐treated bacteria were harvested by centrifuging at 8000 rpm for 3 min and fixed by 2.5% glutaraldehyde after coincubated 2 h. The TBP‐treated bacteria samples for TEM characterization were prepared according to the previous reported procedure.^[^ ^46^ ^]^ The TBPs‐treated bacteria were characterized by TEM (HT7700, Hitachi, Japan).

##### Mechanism of Killing Internalized Bacteria Analysis

IEC‐6 cells were pro‐treated with CQ for 3 h, then IEC‐6 cells were infected with *S. aureus* ATCC 29213 at the MOI of 1 for 4 h, then we discarded the medium and washed the cells with PBS for three times to remove the noninternalizing bacteria. Subsequently, cells were incubated with fresh DMEM containing 100 µg mL^−1^ gentamycin for 15 min, and washed with PBS for three times.^[^ ^18^, ^32^ ^]^ Gentamycin has no effect on killing intracellular *S. aureus* under such condition. Lastly, the cells were incubated in new DMEM in the presence of 1 µg mL^−1^ TBP‐1 for another 2 h, then stained with LC3 (Ex = 552 nm, Em = 565 nm) and DAPI (Ex = 405 nm, Em = 454 nm), the number of internalized *S. aureus* was counted by plate. Also, the number of internalized *S. aureus* was analyzed by FACS. IEC‐6 cells infected with *S. aureus* ATCC 29213 with green fluorescence for different times (0, 2, 4, 8, 12 and 24 h). The data were reanalyzed by setting gates after removed noninternalizing bacteria. IEC‐6 cells were pro‐treated with NAC for 3 h, then IEC‐6 cells were infected with *S. aureus* at the MOI of 1 for 4 h, *S. aureus* were removed, then cells were treated with 1 µg mL^−1^ TBP‐1 for 2 h, last cells were then stained with DAPI (Ex = 405 nm, Em = 454 nm) and F‐actin (Ex = 552 nm, Em = 565 nm), the number of internalized *S. aureus* was counted by plate. For static images, fixed and stained intestinal or cellular samples were captured by a Leica SP8 confocal microscope (Leica, Gremany). 3D images were taken by capture all the *X*‐, *Y*‐, and *Z*‐axis sections. For analyzing the location of internalized bacteria, the *Z*‐axis section was cut every 1 or 2 µm. Images were analyzed and merged by the LAS AF Lite software (Leica, Gremany). Cells were treated as same, and then were tracked by lysotracker red, and cells were observed through confocal microscope. IEC‐6 cells were infected with *S. aureus* at the MOI of 1 for 4 h, *S. aureus* were removed, then cells were treated with 1 µg mL^−1^ TBP‐1 for 2 h. The mitochondria of IEC‐6 were tracked by Mito‐tracker (Ex = 552 nm, Em = 600 nm). The morphological change was observed by confocal microscopy. The ROS of IEC‐6 were tracked by ROS assay kit. ROS change was observed by microplate reader (Tecan, infinite M200, Switzerland) (Ex = 488 nm, Em = 525 nm). Additionally, cytochalasin D was used to explore whether TBP‐1 affects cell phagocytosis. IEC‐6 cells were incubated with cytochalasin D (1 µmol L^−1^) and TBP‐1 (1 µmol L^−1^) for 1 h. Latex beads (1:1000) were added for 1 h, then extracellular latex beads were removed. The fluorescence intensity of GFP was measured by the fluorescence microplate reader, and the fluorescence imagings were recorded by CLSM.

##### Western Blot

IEC‐6 cells were infected with *S. aureus* at the MOI of 1 for 4 h, noninternalizing *S. aureus* were removed as before, then cells were treated with 1 µg mL^−1^ TBP‐1 (1 µg mL^−1^ TBP‐2) for 2 h, then the levels of LC3 and p62 were tested by Western blot. CQ (50 µg mL^−1^)/Rapa (100 × 10^−9^ m) was used to treat IEC‐6 cells before *S. aureus* infected. All proteins were normalized to the level of *β*‐actin. The primary antibodies included rabbit anti‐LC3B, anti‐p62 (Abcam, UK), and mouse anti‐*β*‐actin antibodies (Proteintech, USA), secondary antibodies were goat antirabbit and goat antimouse antibodies (Beyotime, China). Gray values of protein bands were quantified by Image J software.

##### Mitochondrial Membrane Potential Assay

The mitochondrial membrane potential (Δ*Ψ*_m_) in IEC‐6 cells based on the cyanine dye JC‐1 staining assay. JC‐1 aggregates in the mitochondrial matrix to form a polymer, which emits a strong red fluorescence. Due to the decrease or loss of membrane potential in unhealthy mitochondria, JC‐1 can only exist as a monomer in the cytoplasm, producing green fluorescence. IEC‐6 cells were infected with *S. aureus* ATCC 29213 at the MOI of 1 for 4 h treated with or without TBP‐1, IEC‐6 cells treated with TBP‐1, and IEC‐6 cells were stained with JC‐1, respectively. The imaging was captured by a Leica SP8 confocal microscope (Leica, Gremany), and Green and red fluorescence ratio of ICE‐6 cells analyzed by Image J software.

##### Distribution of TBP‐1

The concentrations of extracellular TBP‐1, internalized TBP‐1 in the cytosol of IEC‐6 cells, TBP‐1 in *S. aureus*, and loss antibiotics were quantified by LC–MS/MS.

##### Mouse Sepsis Protection Model

6–8 weeks old female BALB/c mice were purchased from the Vital River Laboratory Animal Technology Co. Ltd. (Beijing, China) and housed under specified pathogen‐free conditions for one week. The in vivo bioavailability of TBP‐1 and TBP‐2 was tested against a clinical isolate MRSA T144 in a mouse sepsis protection model according to a previous protocol. Briefly, seven groups of BALB/c female mice (*n* = 6) were infected intraperitoneally with 0.5 mL of bacterial suspension (7.5 × 10^8^ CFU per mouse) and monitored daily for survival. After 1 h postinfection, mice in each group were treated with TBP‐1 at single intraperitoneal doses of 1.25 and 2.5 mg kg^−1^, while TBP‐2 at dose of 2.5 and 5 mg kg^−1^. Additionally, two groups were treated with vancomycin at doses of 2, 5, and 10 mg kg^−1^ as positive controls.^[^ ^47^ ^]^ For determination of the bacterial burden, mice were sacrificed at 48 h. Heart, liver, spleen, lung, and kidney were collected and homogenized in sterile PBS. Serial dilutions of each suspension were plated on *S. aureus* chromogenic agar plates (CHROM agar) for the enumeration of bacterial colonies.

##### Hematoxylin–Eosin (HE) Staining

Samples of mouse organs were excised and fixed with 4% paraformaldehyde solution (Sigma). Histological images were taken using an inverted microscope (Olympus, IX71, Japan). Histopathological changes were analyzed by a traditional HE staining protocol.

##### Ethics Statement

Animal experiments were performed in strict accordance with the regulations for the Administration of Affairs Concerning Experimental Animals approved by the State Council of People's Republic of China (11‐14‐1988). The animal study protocols were performed in accordance with the relevant guidelines and regulations (ID: SKLAB‐B‐2010‐003). The laboratory animal usage license number is SYXK‐2016‐0008, certified by Beijing Association for Science and Technology.

##### In Vivo Imaging and Toxicity of TBP‐Treated Mice

6–8 weeks old female BALB/c mice were randomly selected for fluorescence imaging experiments. Mice in each group were treated with TBP‐1 at single intraperitoneal doses of 2.5 mg kg^−1^, while TBP‐2 at dose of 5 mg kg^−1^. In vivo fluorescence imaging of main organs (kidney, heart, liver, spleen, and lung) was performed with Maestro EX fluorescence imaging system (CRi, Inc.) after 1, 3, 5, and 10 days postinjection. The light with a central wavelength at 480 nm was selected as the excitation, and fluorescence signal in the spectral region of 670 nm was collected. The fluorescence of TBPs in blood was conducted on a Perkin‐Elmer spectrofluorometer LS 55. Weight was also measured after 1, 3, 5, and 10 days postinjection, respectively.

##### Preparation of Samples In Vitro Systems

The incubation mixture (total volume = 500 µL) consisting of 2 mg protein mL^−1^ of liver microsomes (the volume was 447.5 µL) and 2 × 10^−3^ m NADPH‐generating system (the volume was 50 µL) was preincubated for 5 min at 37 °C. The reaction was initiated there after by the addition of 2.5 µL of TBPs (10 × 10^−3^ m). Incubation mixtures without TBPs and a NADPH‐generating system served as control. After 2 h of incubation at 37 °C in a metabolic shaker, the reaction was terminated by adding 500 µL of ice cold ethyl acetate. After evolution and centrifugation at 12 000 rpm at 4 °C for 15 min, the supernatant was filtered through a 0.22 µm microbore cellulose membrane into an autosampler vial and analyzed by UPLC‐Q/TOF‐MS for identification of metabolites.^[^ ^48^ ^]^

##### Statistical Analysis

The values reported are expressed as mean standard deviation (SD) and were analyzed statistically by the paired Student's *t*‐test method and comparisons among more than two groups were obtained by ANOVA. The Origin 8 software was used for graph plotting. A value of *P* < 0.05 was considered significant and was indicated with asterisks: *P < 0.05 and **P < 0.001, ns not significant and was indicated with asterisks ns > 0.05. The statistical significance of differences between groups was performed by using pair‐sample *t*‐testing method on the Origin 8.0 software. Each experiment included at least three replicates.

## Conflict of Interest

The authors declare no conflict of interest.

## Supporting information

Supporting Information

## Data Availability

Research data are not shared.

## References

[1] K. Kupferschmidt , Science 2016, 352, 758. 27174968 10.1126/science.352.6287.758

[2] Y. Liu , S. Y. Ding , J. Z. Shen , K. Zhu , Nat. Prod. Rep. 2019, 36, 573. 30324212 10.1039/c8np00031j

[3] M. L. Lambert , C. Suetens , A. Savey , M. Palomar , M. Hiesmayr , I. Morales , A. Agodi , U. Frank , K. Mertens , M. Schumacher , M. Wolkewitz , Lancet Infect. Dis. 2011,11, 30. 21126917 10.1016/S1473-3099(10)70258-9

[4] J. Hui , P. T. Dong , L. J. Liang , T. Mandal , J. J. Li , E. R. Ulloa , Y. W. Zhan , S. Jusuf , C. Zong , M. N. Seleem , G. Y. Liu , Q. Cui , J. X. Cheng , Adv. Sci. 2020, 7, 1903117. 10.1002/advs.201903117 PMC7080515 32195102

[5] M. McKenna , https://www.wired.com/2013/09/cdc‐amr‐rpt1/ 2013 (accessed: September 2013).

[6] J. L. Martínez , F. Baquero , D. I. Andersson , Nat. Rev. Microbiol. 2007, 5, 958. 18007678 10.1038/nrmicro1796

[7] G. E. Thwaites , V. Gant , Nat. Rev. Microbiol. 2011, 9, 215. 21297670 10.1038/nrmicro2508

[8] Z. Q. Yang , Y. L. Huang , H. W. Zhou , R. Zhang , K. Zhu , Lancet Infect. Dis. 2017, 18, 22. 29102519 10.1016/S1473-3099(17)30627-8

[9] M. A. Kohanski , D. J. Dwyer , B. Hayete , C. A. Lawrence , J. J. Collins , Cell 2007, 130, 797. 17803904 10.1016/j.cell.2007.06.049

[10] a) C. Walsh , Nat. Rev. Microbiol. 2003, 1, 65; 15040181 10.1038/nrmicro727

[11] a) J. Li , X. M. Liu , L. Tan , Z. D. Cui , X. J. Yang , Y. Q. Liang , Z. Y. Li , S. L. Zhu , Y. F. Zheng , K. W. K. Yeung , X. B. Wang , S. L. Wu , Nat. Commun. 2019, 10, 4490; 31582736 10.1038/s41467-019-12429-6 PMC6776522

[12] a) J. D. Luo , Z. L. Xie , J. W. Y. Lam , L. Cheng , H. Y. Chen , C. F. Qiu , R. T. K. Kwork , X. W. Zhan , Y. Q. Liu , D. B. Zhu , B. Z. Tang , Chem. Commun. 2001, 1740; 10.1039/b105159h 12240292

[13] C. C. Zhou , W. H. Xu , P. B. Zhang , M. J. Jiang , Y. C. Chen , R. T. K. Kwok , M. M. S. Lee , G. G. Shan , R. L. Qi , X. Zhou , J. W. Y. Lam , S. Wang , B. Z. Tang , Adv. Funct. Mater. 2019, 29, 1805986.

[14] B. Situ , X. Y. Ye , Q. W. Zhao , L. Y. Mai , Y. F. Huang , S. Q. Wang , J. Chen , B. Li , B. R. He , Y. Zhang , J. J. Zou , B. Z. Tang , X. H. Pan , L. Zheng , Adv. Sci. 2020, 7, 1902760. 10.1002/advs.201902760 PMC7029725 32099764

[15] Y. Li , Z. Zhao , J. J. Zhang , R. T. K. Kwok , S. Xie , R. B. Tang , Y. X. Jia , J. C. Yang , L. Wang , J. W. Y. Lam , W. F. Zheng , X. Y. Jiang , B. Z. Tang , Adv. Funct. Mater. 2018, 1804632.

[16] C. Dias , J. P. Pais , R. Nunes , M. T. Blazquez , J. T. Marques , A. F. Almeida , P. Serra , N. M. Xavier , D. Vila‐Vicosa , M. Machuqueiro , A. S. Viana , A. Martins , M. S. Santos , A. Pelerito , R. Dias , R. Tenreiro , M. C. Oliveira , M. Contino , N. A. Colabufo , R. F. M. de Almeida , A. P. Rauter , Nat. Commun. 2018, 9, 4857. 30451842 10.1038/s41467-018-06488-4 PMC6242839

[17] X. W. He , L.‐H. Xiong , Z. Zhao , Z. Y. Wang , L. Luo , J. W. Y. Lam , R. T. K. Kwok , B. Z. Tang , Theranostics 2019, 9, 3223. 31244951 10.7150/thno.31844 PMC6567968

[18] F. Hu , G. B. Qi , K. Kenry , D. Mao , S. W. Zhou , M. Wu , W. B. Wu , B. Liu , Angew. Chem., Int. Ed. 2020, 59, 9288. 10.1002/anie.201910187 31449353

[19] C. L. Zhu , Q. Yang , F. T. Lv , L. B. Liu , S. Wang , Adv. Mater. 2013, 25, 1203. 23280674 10.1002/adma.201204550

[20] Y. Liu , S. Y. Ding , R. Dietrich , E. Märtlbauer , K. Zhu , Angew. Chem., Int. Ed. 2017, 56, 1486. 10.1002/anie.201609277 28106348

[21] National Committee for Clinical Laboratory Standards, Methods for determining bactericidal activity of antimicrobial agents, M26‐A, 1999.

[22] X. Y. Zheng , D. Wang , W. H. Xu , S. Q. Cao , Q. Peng , B. Z. Tang , Mater. Horiz. 2019, 6, 2016.

[23] X. J. Shi , H. P. S. Simon , J. H. C. Chau , Y. Li , Z. Y. Liu , R. T. K. Kwok , J. K. Liu , P. H. Xiao , J. J. Zhang , B. Liu , J. W. Y. Lam , B. Z. Tang , Small Methods 2020, 2000046.

[24] J. M. Monteiro1 , P. B. Fernandes1 , F. Vaz , A. R. Pereira1 , A. C. Tavares1 , M. T. Ferreira1 , P. M. Pereira1 , H. Veiga1 , E. Kuru , M. S. VanNieuwenhze , Y. V. Brun , S. R. Filipe , M. G. Pinho1 , Nat. Commun. 2015, 6, 8055. 26278781 10.1038/ncomms9055 PMC4557339

[25] C. Sohlenkamp , O. Geiger , FEMS Microbiol. Rev. 2016, 40, 133. 25862689 10.1093/femsre/fuv008

[26] A. R. Hesser , L. M. Matano , C. R. Vickery , B. M. Wood , A. G. Santiago , H. G. Morris , T. Do , R. Losick , S. Walker , J. Bacteriol. 2020, 202, e00149. 10.1128/JB.00149-20 PMC8404710 32482719

[27] J. J. Foti , B. Devadoss , J. A. Winkler , J. J. Collins , G. C. Walker , Science 2012, 336, 315. 22517853 10.1126/science.1219192 PMC3357493

[28] a) H. Shi , X. Ma , Q. Zhao , B. Liu , Q. Qu , Z. An , Y. L. Zhao , W. Huang , Adv. Funct. Mater. 2014, 24, 4823;

[29] A. M. Cuervo , Trends Cell Biol. 2004, 14, 70. 15102438 10.1016/j.tcb.2003.12.002

[30] a) A. Kuma , M. Matsui , N. Mizushima , Autophagy 2007, 3, 323; 17387262 10.4161/auto.4012

[31] a) L. Harhaji‐Trajkovic , K. Arsikin , T. Kravic‐Stevovic , S. Petricevic , G. Tovilovic , A. Pantovic , N. Zogovic , B. Ristic , K. Janjetovic , V. Bumbasirevic , V. Trajkovic , Pharm. Res. 2012, 29, 2249; 22538436 10.1007/s11095-012-0753-1

[32] X. Y. Liu , F. Liu , S. Y. Ding , J. Z. Shen , K. Zhu , Adv. Sci. 2020, 7, 1900840. 10.1002/advs.201900840 PMC7509632 32999821

[33] M. B. Mestre , C. M. Fader , C. Solar , M. I. Colombo , Autophagy 2010, 6, 110. 20110774 10.4161/auto.6.1.10698

[34] T. Feldkamp , A. Kribben , J. M. Weinberg , Am. J. Physiol. Renal. Physiol. 2015, 288, F1092. 10.1152/ajprenal.00443.2004 15625081

[35] Q. Yang , M. Li , O. B. Spiller , D. O. Andrey , P. Hinchliffe , H. Li , C. Maclean , P. Niumsup , L. Powell , M. Pritchard , A. Papkou , Y. B. Shen , E. Portal , K. Sands , J. Spencer , U. Tansawai , D. Thomas , S. L. Wang , Y. Wang , J. Z. Shen , T. Walsh , Nat. Commun. 2017, 8, 2054. 29233990 10.1038/s41467-017-02149-0 PMC5727292

[36] a) M. A. Bhat , M. Imran , S. A. Khan , N. Siddiqui , Indian J. Pharm. Sci. 2005, 67, 151;

[37] E. G. Zhao , Y. L. Chen , H. Wang , S. J. Chen , J. W. Y. Lam , C. W. T. Leung , Y. N. Hong , B. Z. Tang , ACS Appl. Mater. Interfaces 2015, 7, 7180. 25789982 10.1021/am509142k

[38] J. J. Chen , M. Gao , L. Wang , S. W. Li , J. C. He , A. J. Qin , L. Ren , Y. J. Wang , B. Z. Tang , ACS Appl. Mater. Interfaces 2018, 10, 11436. 29564898 10.1021/acsami.7b18221

[39] S. H. E. Kaufmann , A. Dorhoi , R. S. Hotchkiss , R. Bartenschlager , Nat. Rev. Drug Discovery 2018, 17, 35. 28935918 10.1038/nrd.2017.162 PMC7097079

[40] S. Tiberi , N. du Plessis , G. Walzl , M. J. Vjecha , M. Rao , F. Ntoumi , S. Mfinanga , N. Kapata , P. Mwaba , T. D. McHugh , G. Ippolito , G. B. Migliori , M. J. Maeurer , A. Zumla , Lancet Infect. Dis. 2018, 18, e183. 10.1016/S1473-3099(18)30110-5 29580819

[41] CLSI, *Performance Standards for Antimicrobial Susceptibility Testing*, 28th ed., CLSI Supplement M100, Clinical and Laboratory Standards Institute, Wayne, PA, USA 2018.

[42] W. L. Garner , Plast. Reconstr. Surg. 1998, 102, 135. 9655418 10.1097/00006534-199807000-00021

[43] M. R. Song , Y. Liu , X. Y. Huang , S. Y. Ding , K. Zhu , Nat. Microbiol. 2020, 5, 1040. 32424338 10.1038/s41564-020-0723-z

[44] S. Heeb , M. P. Fletcher , S. R. Chhabra , S. P. Diggle , P. William , M. Camara , FEMS Microbiol. Rev. 2011, 35, 247. 20738404 10.1111/j.1574-6976.2010.00247.x PMC3053476

[45] H. Hamamoto , M. Urai , K. Ishii , J. Yasukawa , A. Paudel , M. Murai , T. Kaji , T. Kuranaga , K. Hamase , T. Katsu , J. Su , T. Adachi , R. Uchida , H. Tomoda , M. Yamada , M. Souma , H. Kurihara , M. Inoue , K. Sekimizu , Nat. Chem. Biol. 2015, 11, 127. 25485686 10.1038/nchembio.1710

[46] Y. Y. Zhao , Y. Tian , Y. Cui , W. W. Liu , W. S. Ma , X. Y. Jiang , J. Am. Chem. Soc. 2010, 132, 12349. 20707350 10.1021/ja1028843

[47] P. Vergidis , M. S. Rouse , G. Euba , M. J. Karau , S. M. Schmidt , J. N. Mandrekar , J. M. Steckelberg , R. Patel , Antimicrob. Agents Chemother. 2011, 55, 1182. 21189340 10.1128/AAC.00740-10 PMC3067063

[48] S. P. Yang , Y. S. Li , X. Y. Cao , D. F. Hu , Z. H. Wang , Y. Wang , J. Z. Shen , S. X. Zhang , J. Agric. Food Chem. 2013, 61, 9734. 23971727 10.1021/jf4012054

