# Supplementary material for: Efficient Killing of Multidrug‐Resistant Internalized Bacteria by AIEgens In Vivo
Source: Adv Sci (Weinh). 2021 Mar 2;8(9):2001750. doi: 10.1002/advs.202001750 (PMC8097328; doi:10.1002/advs.202001750)
Supplement: Supplementary file 1 — Supporting Information [file ADVS-8-2001750-s001.pdf]

WILEY-VCH

© 2021 Wiley-VCH GmbH

ADVANCED SCIENCE
Open Access

Supporting Information

for *Adv. Sci.*, DOI: 10.1002/advs.202001750

Efficient Killing of Multidrug-Resistant Internalized Bacteria by AIEgens *in Vivo*

Ying Li, [†] *Fei Liu,* [†] *Jiangjiang Zhang, Xiaoye Liu, Peihong Xiao, Haotian Bai, Shang Chen, Dong Wang, Simon H. P. Sung, Ryan T. K. Kwok, Jianzhong Shen, Kui Zhu,* Ben Zhong Tang**

Supporting Information

Efficient Killing of Multidrug-Resistant Internalized Bacteria by AIEgens *in Vivo*

Ying Li, [†] *Fei Liu,* [†] *Jiangjiang Zhang, Xiaoye Liu, Peihong Xiao, Haotian Bai, Shang Chen, Dong Wang, Simon H. P. Sung, Ryan T. K. Kwok, Jianzhong Shen, Kui Zhu,* Ben Zhong Tang**

Dr. Y. Li, Dr. P. H. Xiao, Dr. D. Wang, Prof. B. Z. Tang
Center for AIE Research, College of Materials Science and Engineering, Shenzhen University, Shenzhen 518061, China

Dr. Y. Li, F. Liu, Dr. X. Y. Liu, S. Chen, Prof. J. Z. Shen, Prof. K. Zhu
National Center for Veterinary Drug Safety Evaluation, College of Veterinary Medicine, China Agricultural University, Beijing, China, No. 2 Yuanmingyuan West Rd, Beijing 100193, China

E-mail: zhuk@cau.edu.cn

Dr. J. J. Zhang

Department of Biomedical Engineering, Southern University of Science and Technology, No. 1088 Xueyuan Rd, Nanshan District, Shenzhen, 518055, China

Dr. H. T. Bai, Dr. S. H. P. Sung, Dr. R. T. K. Kwok, Prof. B. Z. Tang

Department of Chemistry, Hong Kong Branch of Chinese National Engineering Research Center for Tissue Restoration and Reconstruction, Institute for Advanced Study, Division of Life Science, The Hong Kong University of Science and Technology, Clear Water Bay, Kowloon, Hong Kong, China

E-mail: tangbenz@ust.hk

[†] These authors contributed equally to this work.

Table S1. MICs of TBP-1 and TBP-2 (μg mL^{-1}).

Organisms	Antibiotic resistance genes	Dark		Light	
		TBP-2	TBP-1	TBP-2	TBP-1
S. aureus ATCC 29213	reference strain	0.5	0.25	0.25	0.0625
MRSA QDEF2	*mecA*	1.0	0.5	0.5	0.25
MRSA T144	*mecA*	1.0	0.5	0.5	0.5
E. faecalis 8-3	*optrA*	2.0	1.0	1.0	0.5
E. faecium 4w-9	*optrA*, *cfr*	0.5	1.0	0.25	0.0625
E. faecium VRE-4	*vanA*	0.25	0.0625	0.125	0.0625
E. faecalis VRE-11	*vanA*	0.25	0.125	0.125	0.0625

Bacterial growth in MHB medium (n = 3).

Table S2. MICs of TBP-1 and TBP-2 against *S. aureus* in the presence of CL, PG, PE, LTA and Peptidoglycan (μg mL^{-1}).

AIEgen	Control	CL	PG	PE	LTA	Peptidoglycan
TBP-1	0.25	>16	8	0.5	0.5	1
TBP-2	0.5	16	16	0.5	1	1

CL (Cardiolipin), PG (L-a-phosphatidylglycerol), PE (L-a-phosphatidylethanolamine), LTA (Lipoteichoic acid).

Table S3. MICs of AIEgen (μg mL^{-1}). Bacterial growth in DMEM medium (n = 3)

AIEgen	*S. aureus* ATCC 29213		MRSA QDEF2		MRSA T144	
	Dark	Light	Dark	Light	Dark	Light
TBP-2	1	0.25	1	0.25	1	0.25
TBP-1	0.25	0.0625	0.25	0.125	0.125	0.0615

Table S4. MBC of AIEgen (μg mL^{-1}). Bacterial growth in DMEM medium (n = 3)

AIEgen	*S. aureus* ATCC 29213		MRSA QDEF2		MRSA T144	
	Dark	Light	Dark	Light	Dark	Light
TBP-2	4	0.5	4	0.5	2	1
TBP-1	1	0.125	1	0.125	0.25	0.125

Table S5. IC_{50} ($\mu g\ mL^{-1}$) of AIEgen in different cell lines.

Cell lines	Dark		Light	
	TBP-2	TBP-1	TBP-2	TBP-1
IEC-6	>32	12.33	8.470	1.553
A549	>32	>32	4.405	2.803
HUVEC	>32	19.95	1.972	2.020
3T3	>32	11.91	5.782	3.244

Table S6. Parameters for mass spectrometry analysis of AIEgen

AIEgen	Precursor ion (m/z)	Quan/Qual (m/z)	Collision energy (eV)
TBP-2	278.5	456.1*/248.3	17/16
TBP-1	485.0	456.1*/380.1	36/58

* quantitative ions

Table S7. Full name and source of the cells used in this study.

Cell type	Description	Source / Reference
A549	Lung carcinoma cell	ATCC CRM-CCL-185
IEC-6	Rat small intestine cell	ATCC CRL-1592
NIH/3T3	Mouse embryonic cell	ATCC CRL-1658
HUVEC	Human umbilical vein endothelial cell	ATCC CRL-1730

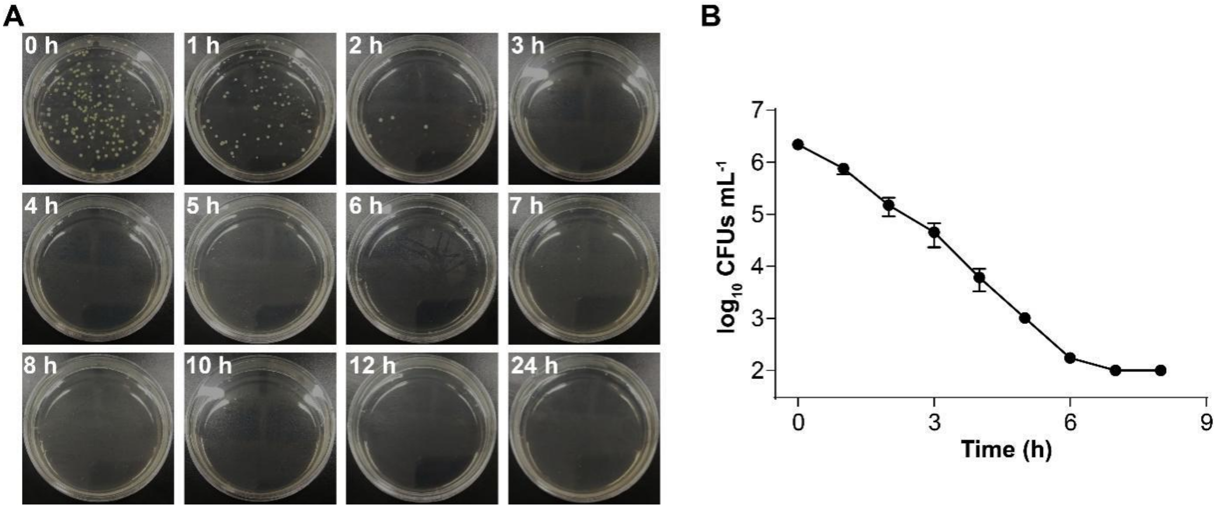

Figure S1. Photographs (A) and CFUs (B) of *S. aureus* cultured on agar plate supplemented with TBP-2 (10 × MICs, 5 µg mL^{-1}) as a function of time in darkness.

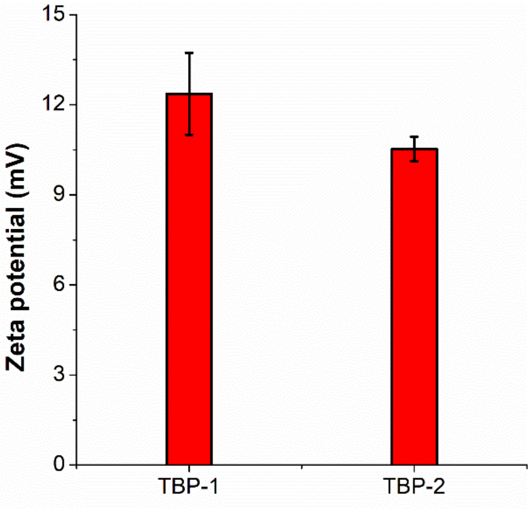

Figure S2. Zeta potential of TBP-1 (5 µg mL^{-1}) and TBP-2 (5 µg mL^{-1}).

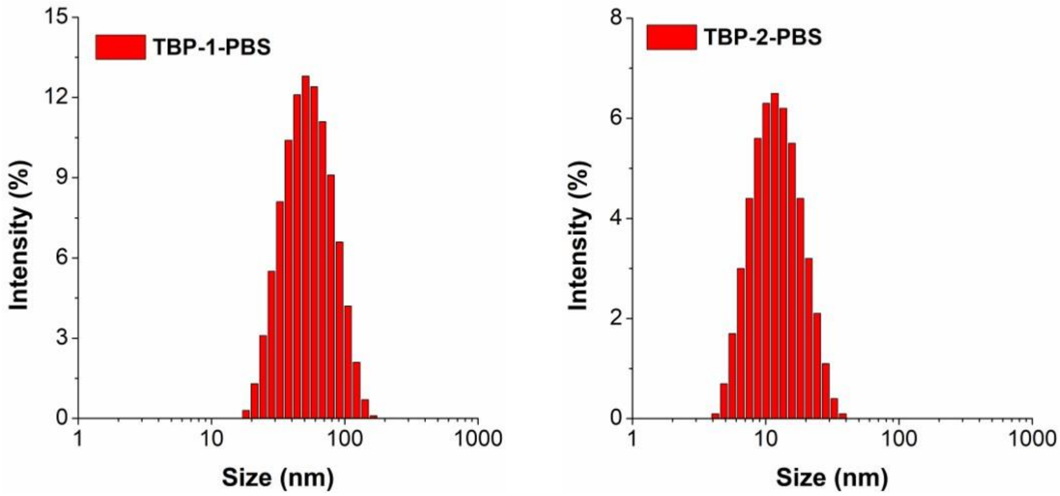

Figure S3. The hydrodynamic diameter distribution of TBP-1 (5 μg mL^{-1}) and TBP-2 (1 μg mL^{-1}) in PBS (pH = 7.4) was measured by DLS.

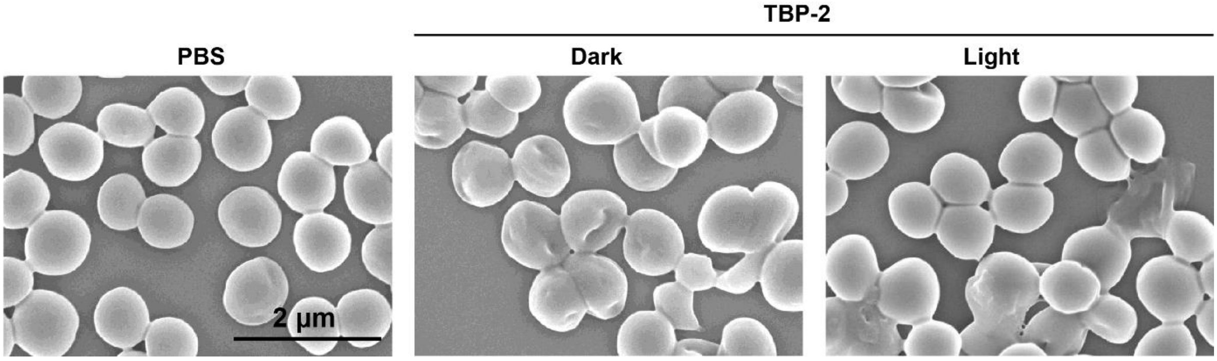

Figure S4. Morphology of *S. aureus* incubated with TBP-2 (dark, 1 μg mL^{-1}; light, 0. 25 μg mL^{-1}) with or without light irradiation (4 mW cm^{-2}), bacteria without treatment were set as control.

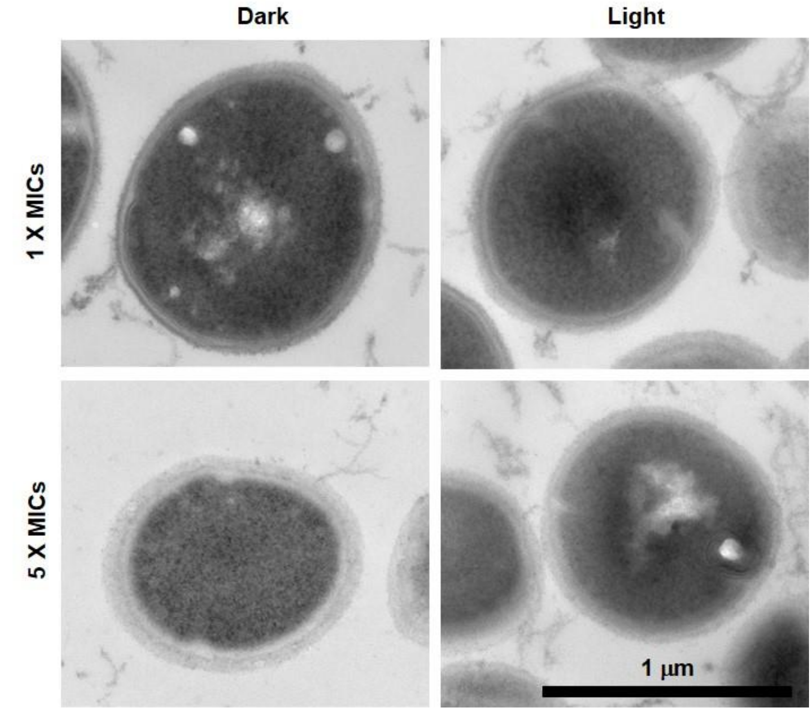

Figure S5. Visualizing TBP-1-induced morphological changes of *S. aureus* with or without white light irradiation (dark, 1 × MICs, 0.25 µg mL^{-1}; 5 × MICs, 1.25 µg mL^{-1}; light, 1 × MICs, 0.0625 µg mL^{-1}; 5 × MICs, 0.3125 µg mL^{-1}) (4 mW cm^{-2}) were imaged by TEM.

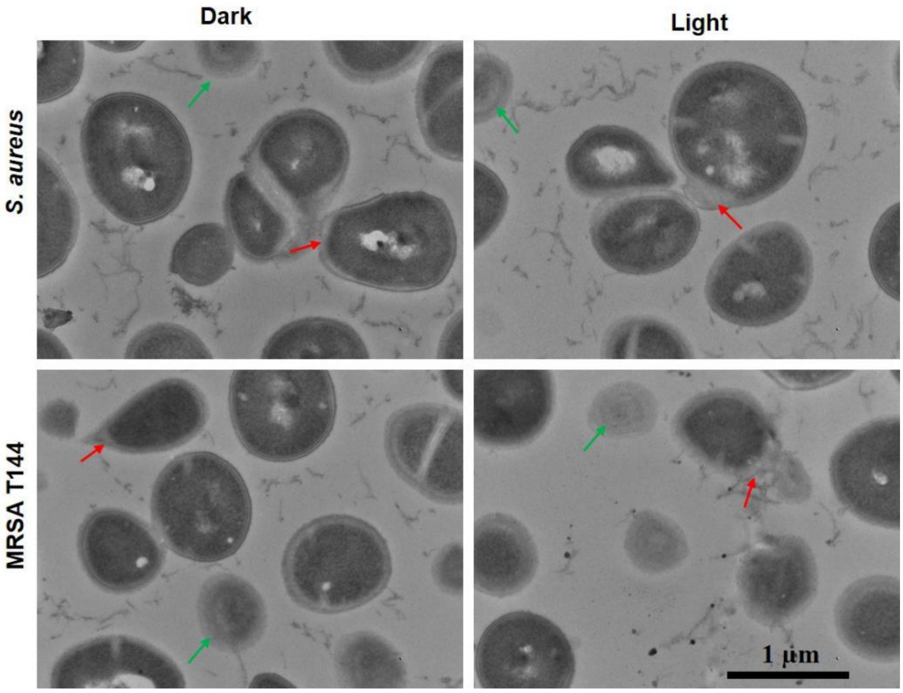

Figure S6. Visualizing TBP-1-induced morphological changes of *S. aureus* (dark, 5 × MICs, 1.25 µg mL^{-1}; light, 5 × MICs, 0.3125 µg mL^{-1}) and MRSA (dark, 10 × MICs, 5 µg mL^{-1}; light, 10 × MICs, 5 µg mL^{-1}) with or without white light irradiation (4 mW cm^{-2}) were imaged by TEM. The red and green arrows represent bacteria with collapsed and lysed structure, respectively.

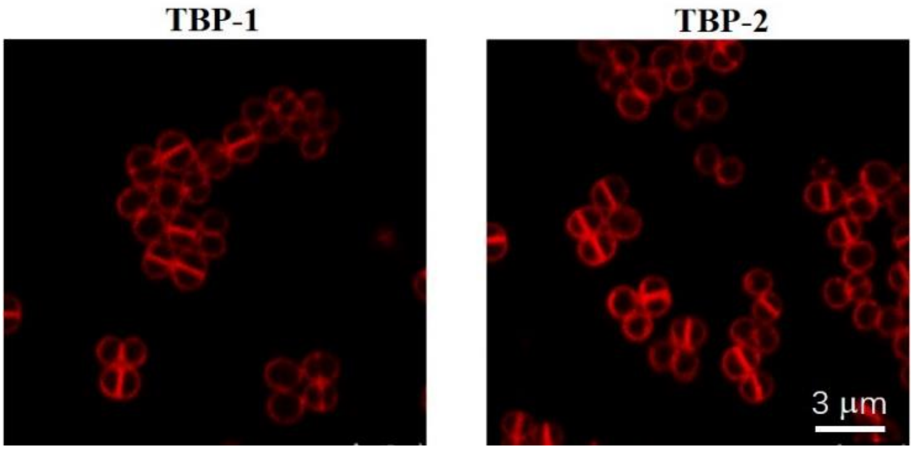

Figure S7. Stimulated emission of depletion microscopy (STED) images of *S. aureus* incubation with TBP-1 (1 μg mL^{-1}) and TBP-2 (1 μg mL^{-1}) for 5 min.

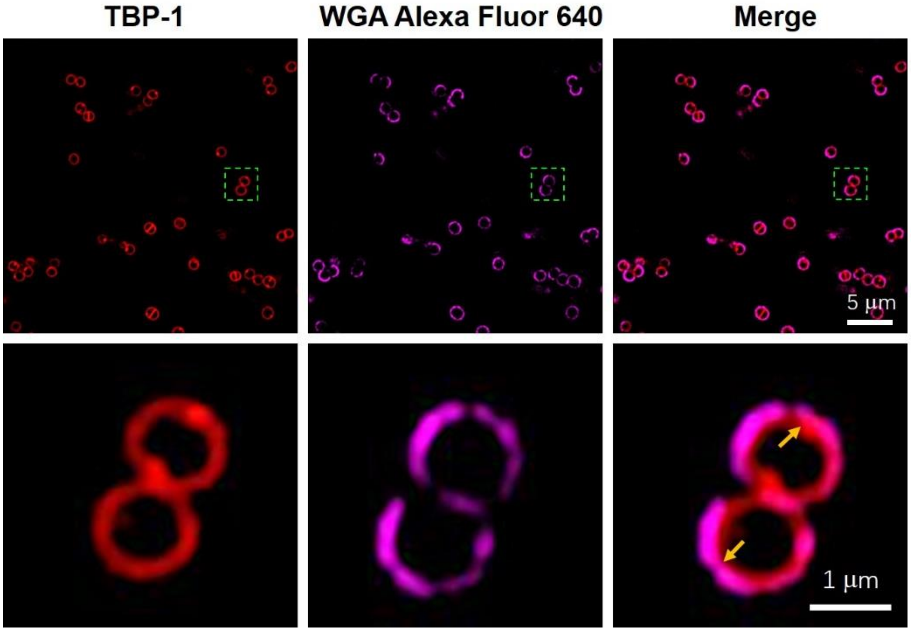

Figure S8. SIM images of *S. aureus* incubated with TBP-1 (1 µg mL^{-1}) and cell wall staining fluorescent dye WGA Alexa Fluor 640 (5 µg mL^{-1}) for 10 min. Magnification images of the outlined area are shown at the bottom, the yellow arrows indicate the co-staining of TBP-1 and WGA Alexa Fluor 640.

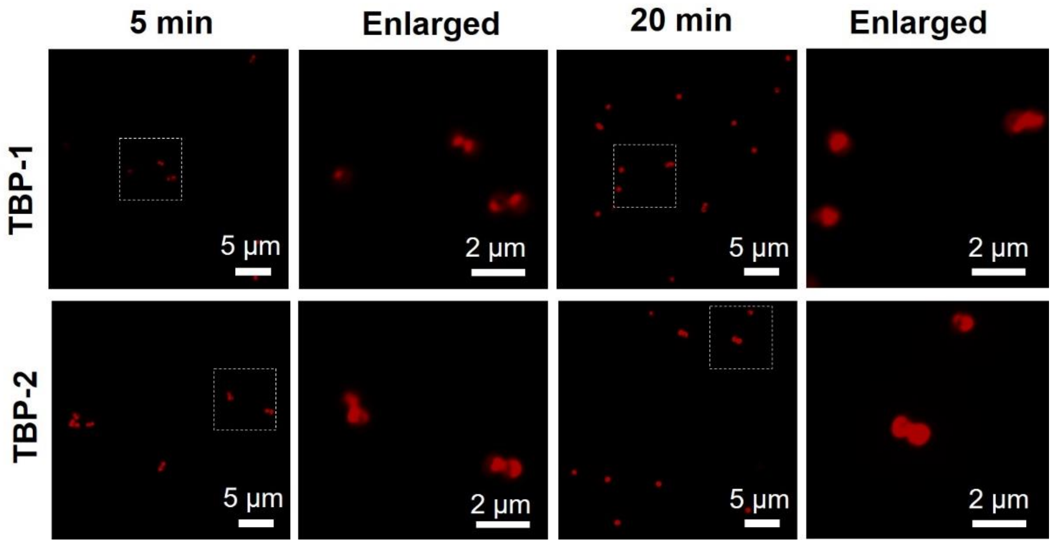

Figure S9. Confocal fluorescent images of *S. aureus* incubated with (2 µg mL^{-1}) of TBP-1 and TBP-2 for 5 min and 20 min. Magnified images of the outlined areas are shown in the right panel.

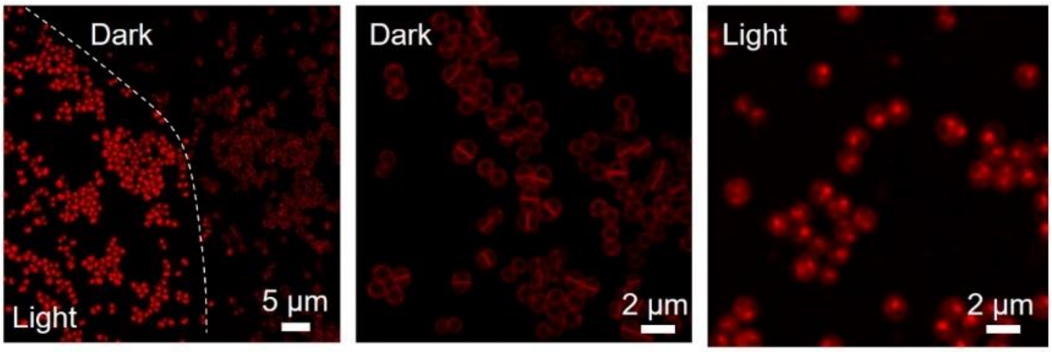

Figure S10. Fluorescence images of *S. aureus* incubated with TBP-1 (1 µg mL^{-1}, 5 min) with or without light irradiation.

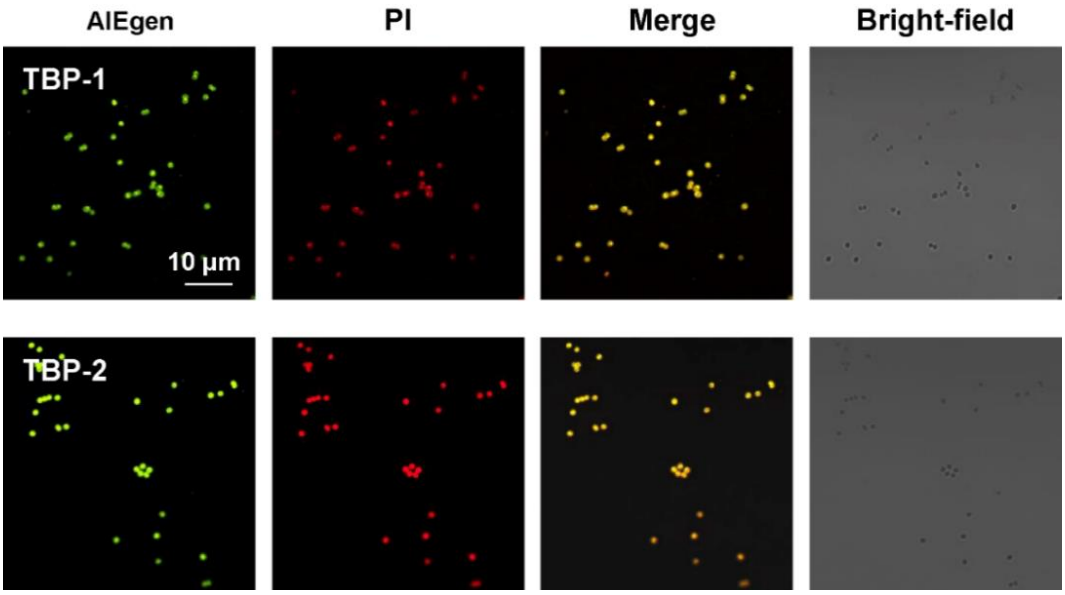

Figure S11. TBP-1 and TBP-2 disrupted the bacterial membrane of *S. aureus*. Images were captured by confocal microscopy. *S. aureus* ATCC 29213 cells were treated with TBP-1 (10 × MICs, 2.5 μg mL^{-1}) and TBP-2 (10 × MICs, 5 μg mL^{-1}) for 10 min, and then incubated with (10 μmol L^{-1}) propidium iodide (PI).

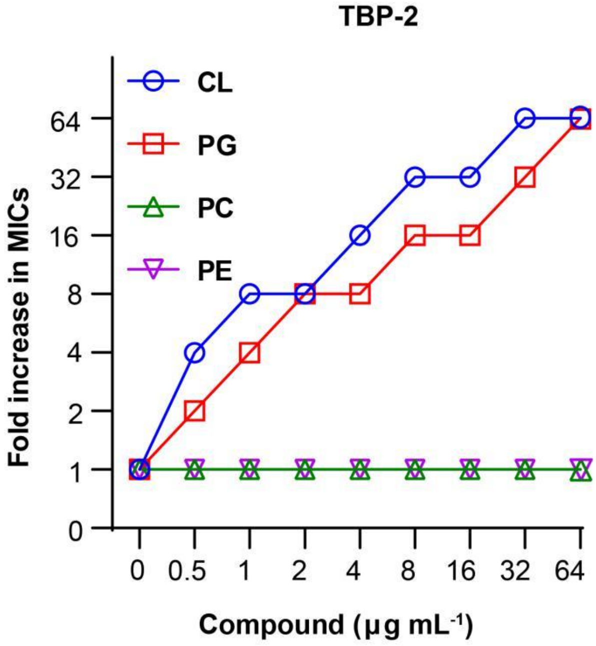

Figure S12. Increased MICs of TBP-2 against *S. aureus* in the presence of CL, PG, PC and PE, ranging from 0 μg mL^{-1} to 64 μg mL^{-1}.

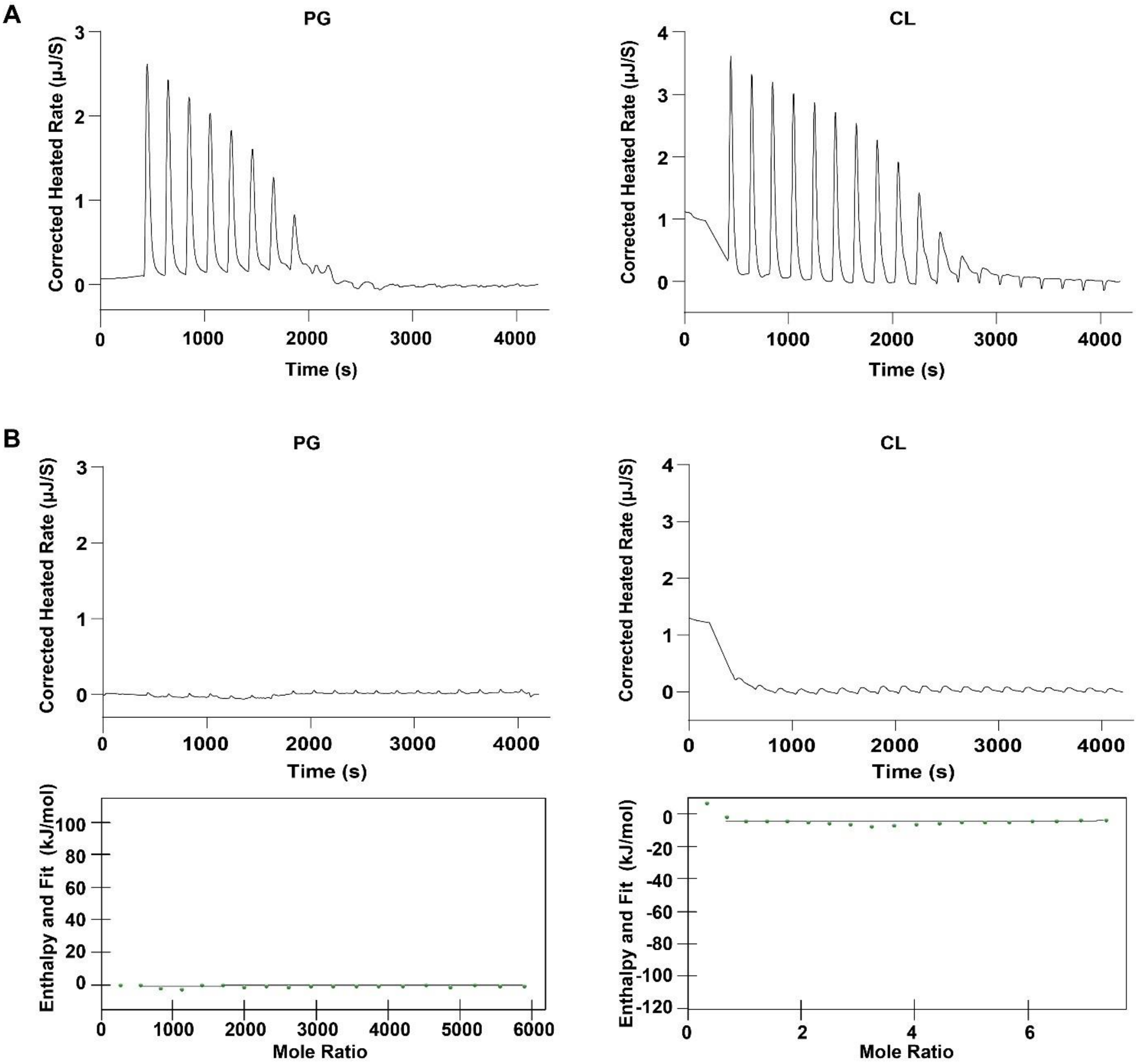

Figure S13. ITC analysis of the interaction between TBP-1 and PG/CL. A) 2 mmol L^{-1} POPG was dropped into 0.5 mmol L^{-1} TBP-1 in HEPES buffer at 25 °C; 0.5 mmol L^{-1} CL was dropped into 0.5 mmol L^{-1} TBP-1 in 10 % alcohol at 25 °C. B) ITC analysis of the interaction between PG/CL and buffer.

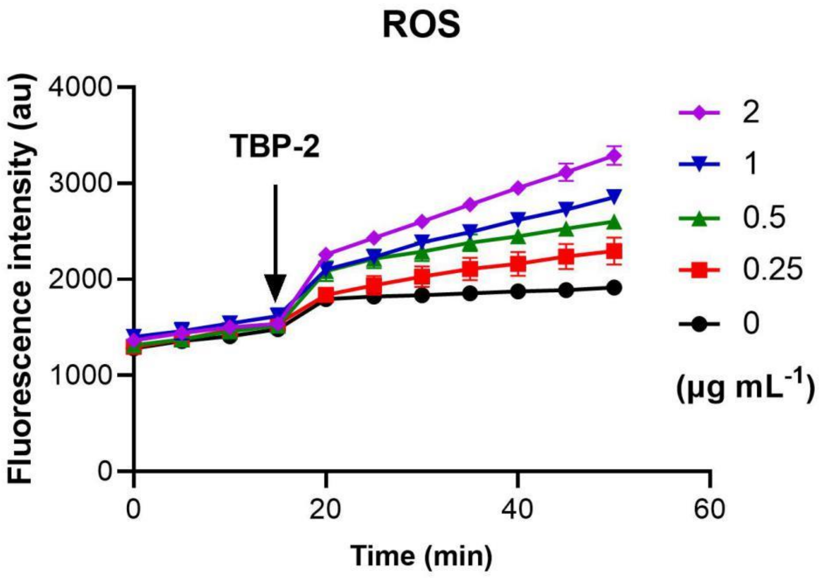

Figure S14. Plot of the internalized ROS fluorescence intensity of *S. aureus* after incubation with TBP-2 (0, 0.25, 0.5, 1 and 2 μg mL^{-1}) as a function of time.

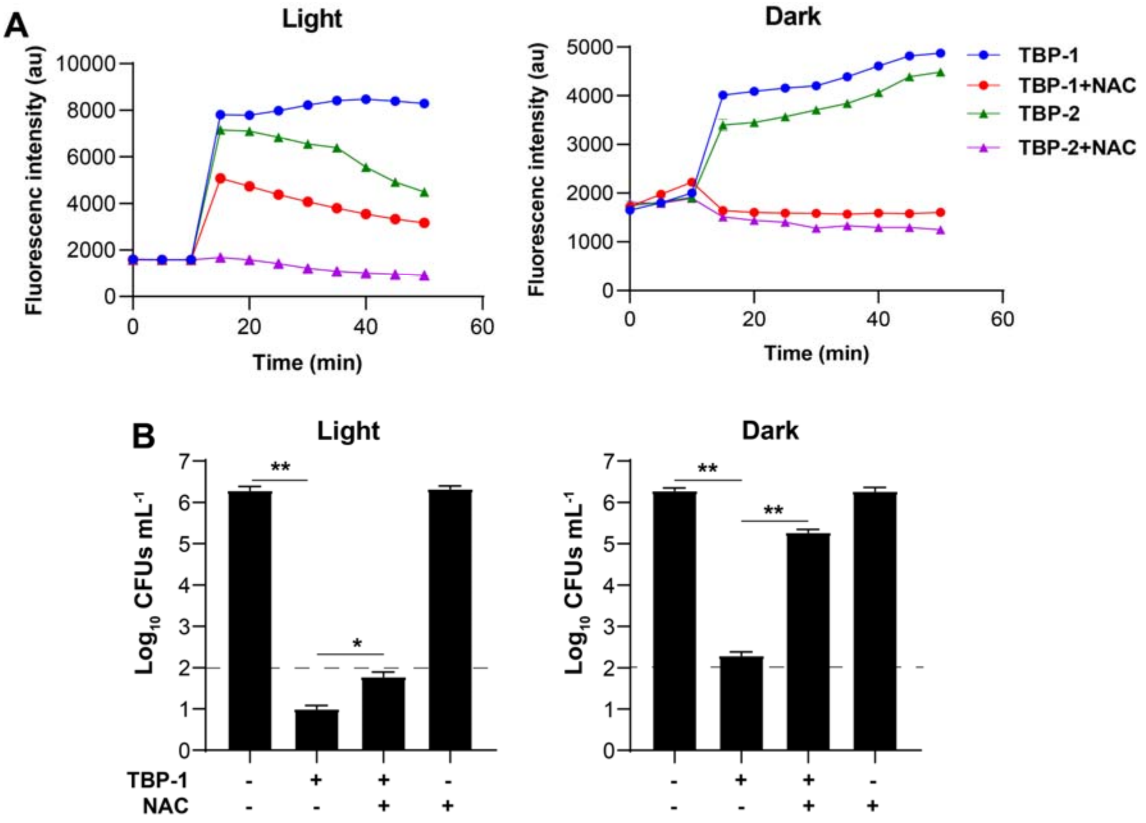

Figure S15. Effects of ROS on antibacterial activity. A) Fluorescence intensity of intracellular ROS in *S. aureus* after the incubation with TBP-1 and TBP-2 in the presence or absence of NAC (50 μmol L^{-1}), under the darkness and light irradiation as a function of time. B) Antibacterial activity of TBPs. The dashed line is the lowest detection line. (**P* < 0.05; ***P* < 0.01, *ns* > 0.05, n = 3).

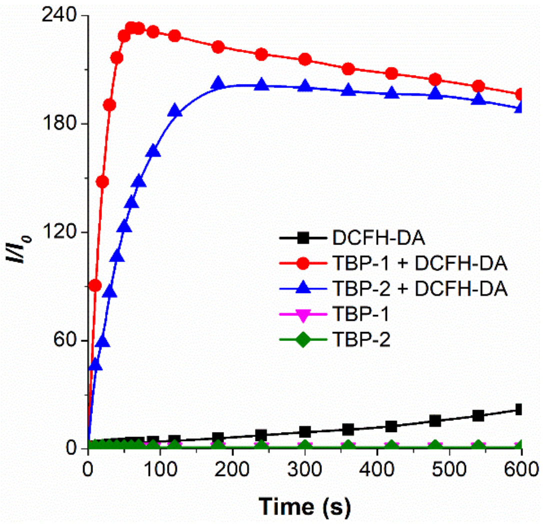

Figure S16. Plotting of relative fluorescence intensity at 525 nm generation by TBP-1 (1 µg mL^{-1}) and TBP-2 (1 µg mL^{-1}) versus the irradiation time with or without DCFH-DA (40 µmol L^{-1}).

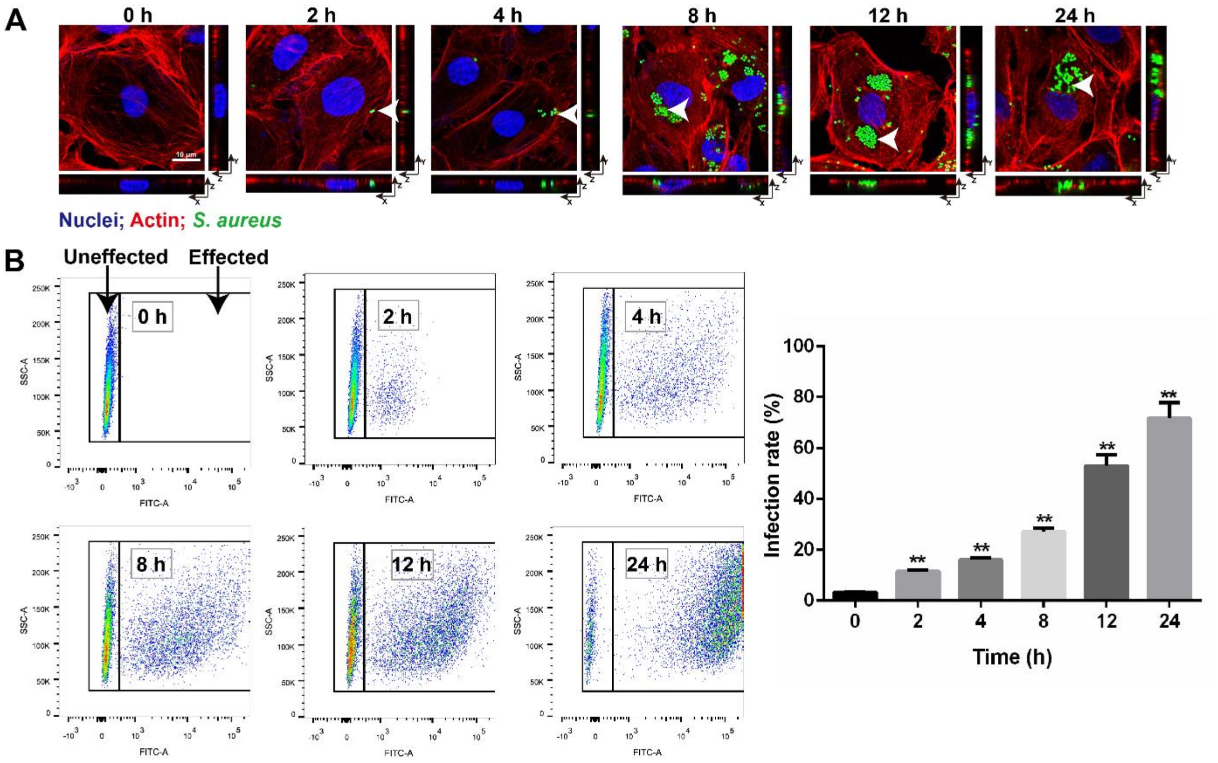

Figure S17. *S. aureus* invaded IEC-6 cells in time dependent manner. IEC-6 cells were infected with GFP *S. aureus* ATCC 29213 for different time (0 h, 2 h, 4 h, 6 h, 8 h, 12 h, 24 h). A) Representative images of *S. aureus* infected IEC-6 cells. B) Infection rate of IEC-6 cells was examined by flow cytometry analysis. (**P <0.001, n = 3).

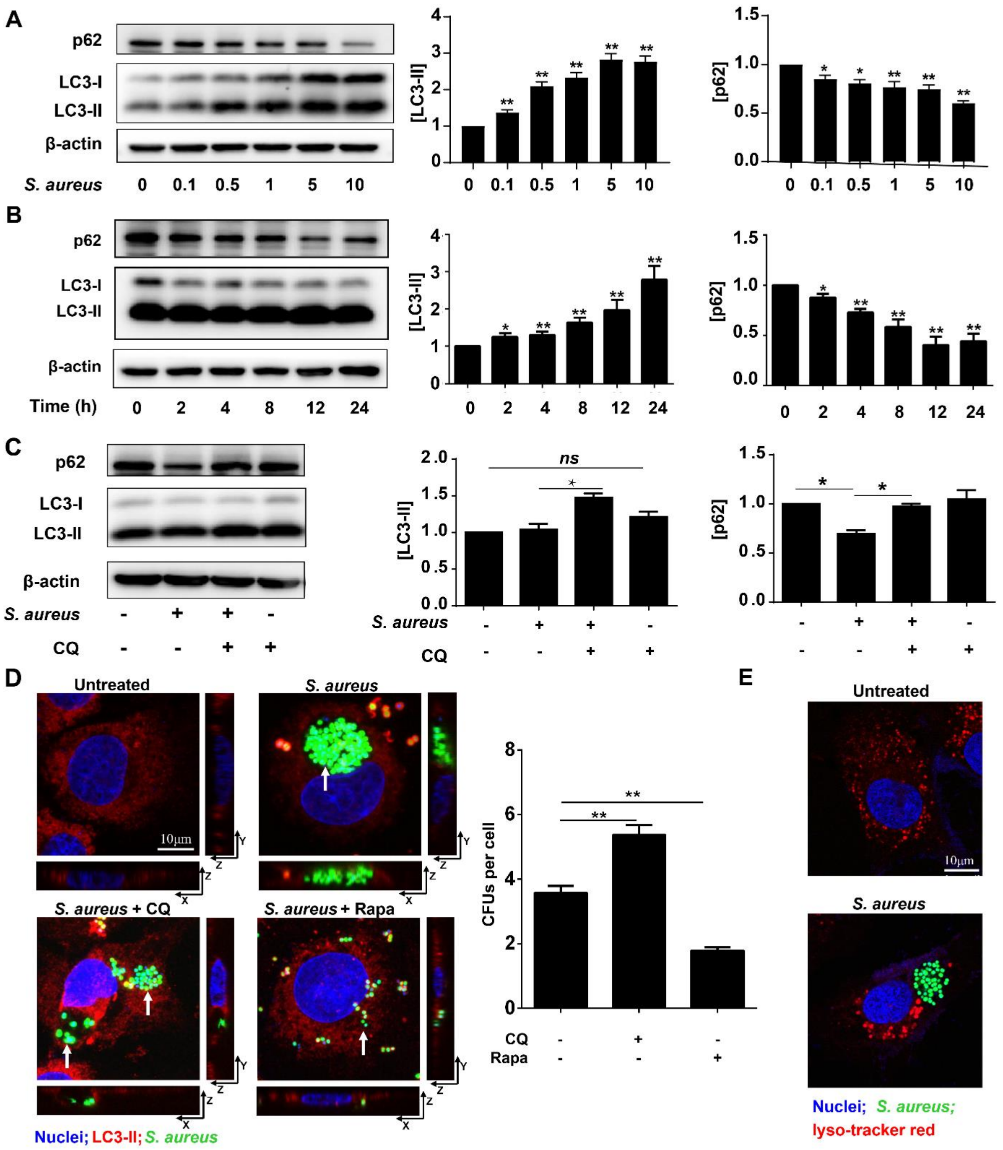

Figure S18. *S. aureus* lived in IEC-6 cells through hijacking autophagy. Western blot analysis of Microtubule Light Chain 3 (LC3) and p62 expression in IEC-6 cells infected with *S. aureus*. A) IEC-6 cells were infected with *S. aureus* ATCC 29213 at different MOI for 4 h. B) IEC-6 cells were infected with *S. aureus* at the MOI=1 for different time. C) CQ was used as a positive control to test autophagy. All proteins were normalized to the level of β-actin. D) Fluorescent images and CFUs of internalized *S. aureus* after IEC-6 cells incubated with *S. aureus, S. aureus* + CQ and *S. aureus* + Rapa. IEC-6 cells were stained with LC3-Ⅱ (Ex = 552 nm, Em = 565 nm) and DAPI (Ex = 405 nm, Em = 454 nm), $^{**}P$ <0.001. E) Fluorescence images of ICE-6 cells incubated with or without *S. aureus*, and then stained with lysotracker red and DAPI.

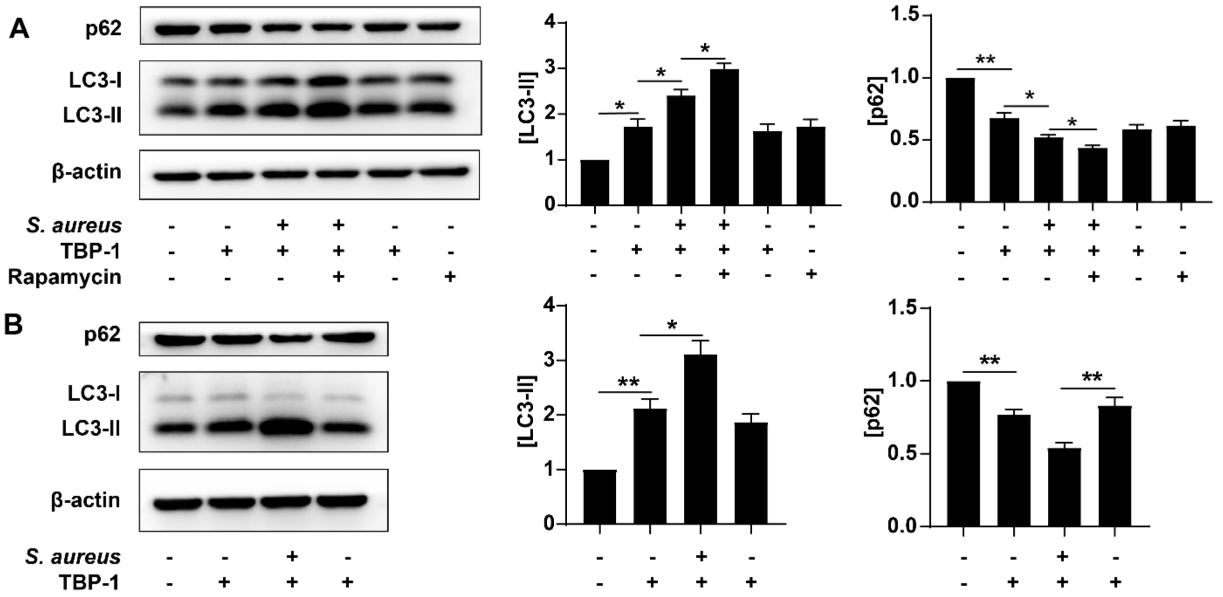

Figure S19. TBP-1 enhanced autophagy. A) Rapamycin (100 nmol L^{-1}) was used as a positive control to analyze autophagy. B) IEC-6 cells was infected with *S. aureus* ATCC 29213, and then treated with TBP-1 (0.25 µg mL^{-1}) in light irradiation. (*$P < 0.05$; **$P < 0.01$, *ns* > 0.05, n = 3).

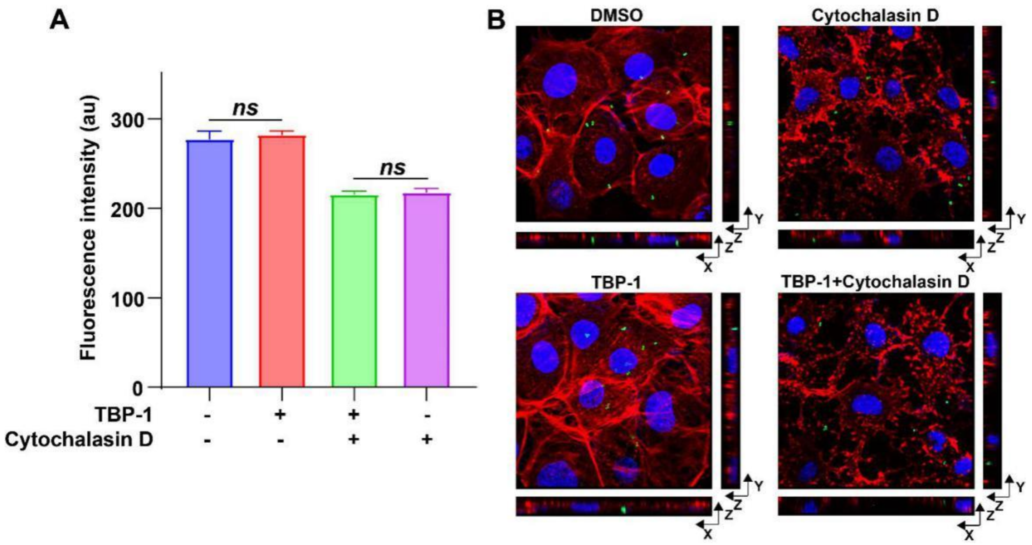

Figure S20. TBP-1 has no significant effect on phagocytosis. A) IEC-6 cells were incubated with cytochalasin D (1 μmol L^{-1}) and TBP-1 (1 μmol L^{-1}) for 1 h. Latex beads (1:1000) were added for 1 h, then extracellular latex beads were removed. The fluorescence intensity of GFP was measured by the fluorescence microplate reader. B) Fluorescent images of IEC-6 cells.

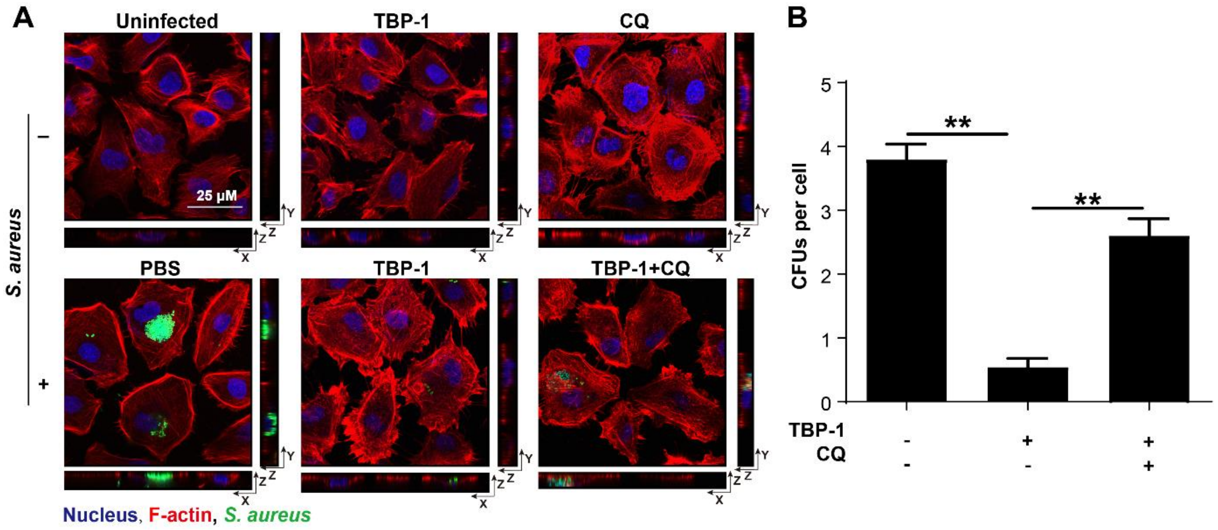

Figure S21. Autophagy was involved in the TBP-1-induced *S. aureus* death. Fluorescent images (A) and CFUs (B) of internalized *S. aureus* after IEC-6 cells incubated with *S. aureus*, *S. aureus* + TBP-1 and a lysosome inhibitor CQ + *S. aureus* + TBP-1. IEC-6 cells were stained with DAPI (Ex = 405 nm, Em = 454 nm) and F-actin (Ex = 552 nm, Em = 565 nm), $^{**}P$ <0.001.

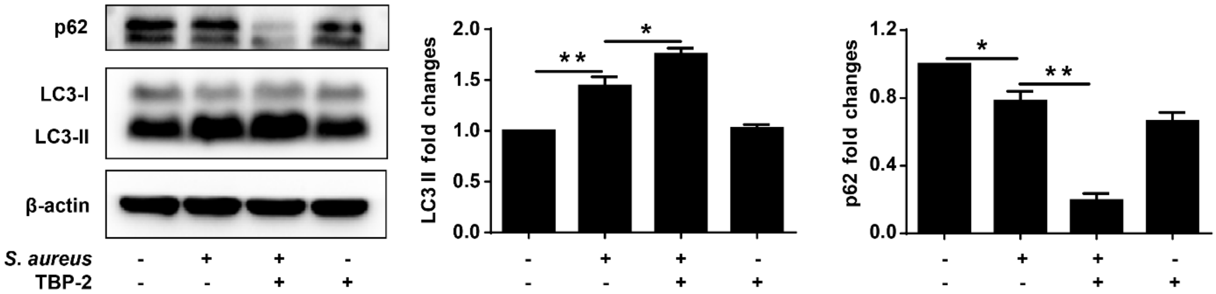

Figure S22. Western blot analysis of LC3 and p62 expression in IEC-6 cells infected with or without *S. aureus* in the presence and absence of TBP-2. All proteins were normalized to the level of β-actin, **P* <0.05, ***P* <0.001.

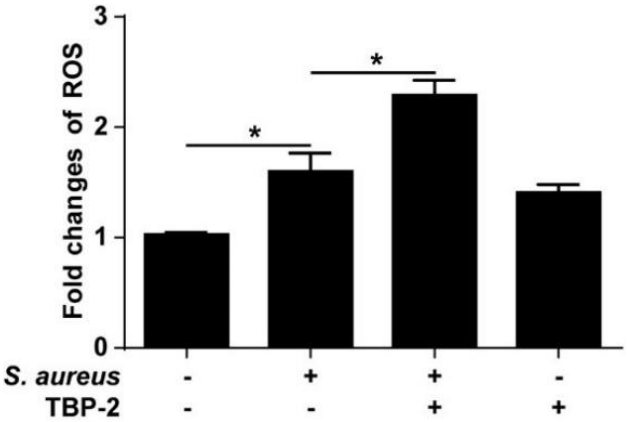

Figure S23. Fold changes of ROS in IEC-6 cells infected with *S. aureus* in the presence and absence of TBP-2 (*P <0.05).

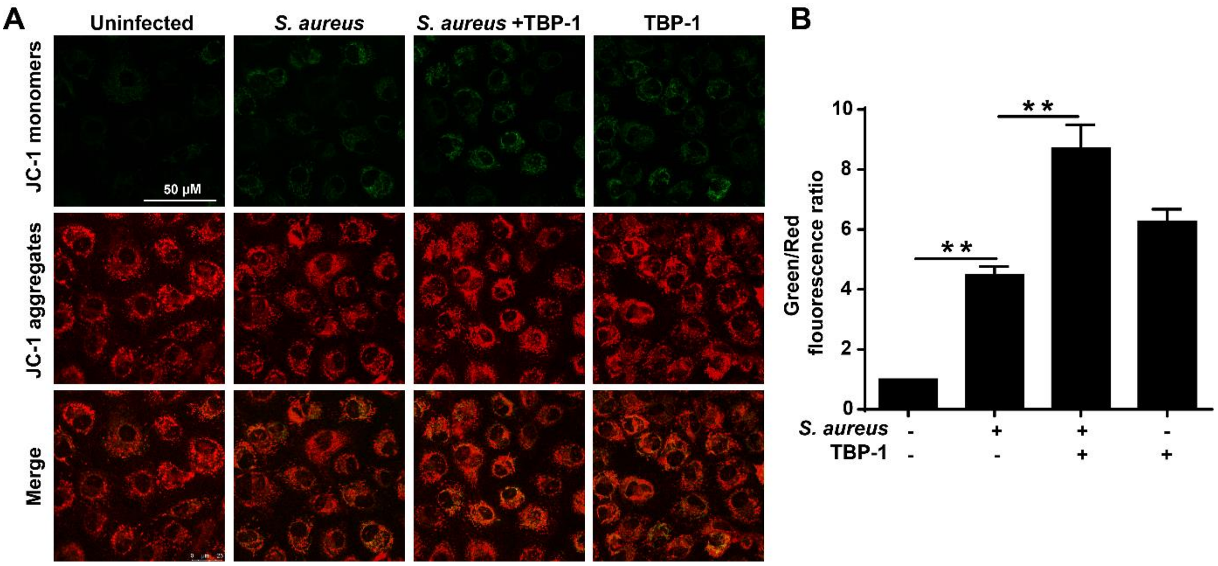

Figure S24. TBP-1 destroyed mitochondrial membrane potential (Δψm). A) Δψm in IEC-6 cells or IEC-6 cells incubation with *S. aureus*, *S. aureus* + TBP-1 and TBP-1 was determined by JC-1 staining. JC-1 aggregates in the mitochondrial matrix to form a polymer, which emits a strong red fluorescence (Ex = 552 nm, Em = 590 nm). Due to the decrease or loss of membrane potential in unhealthy mitochondria, JC-1 can only exist as a monomer in the cytoplasm, producing green fluorescence (Ex = 488 nm, Em = 529 nm). B) Green/red fluorescence ratio of ICE-6 cells after different treatments indicated. $*P < 0.05$, $**P < 0.001$.

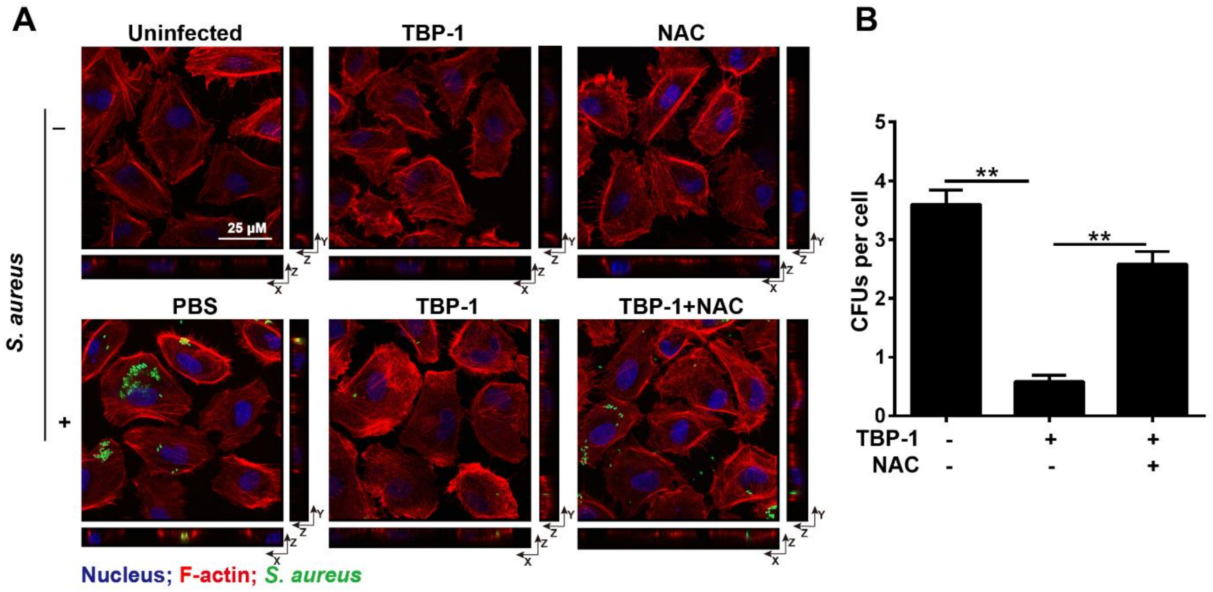

Figure S25. TBP-1 killed internalized *S. aureus*. Fluorescent images (A) and CFUs (B) of internalized *S. aureus* after IEC-6 cells incubated with *S. aureus* at the MOI=1, *S. aureus* + TBP-1 (2 µg mL^{-1}) and NAC (5 mmol L^{-1}) + *S. aureus* + TBP-1. IEC-6 cells were stained with DAPI (Ex = 405 nm, Em = 454 nm) and F-actin (Ex = 552 nm, Em = 565 nm), ***P* <0.001.

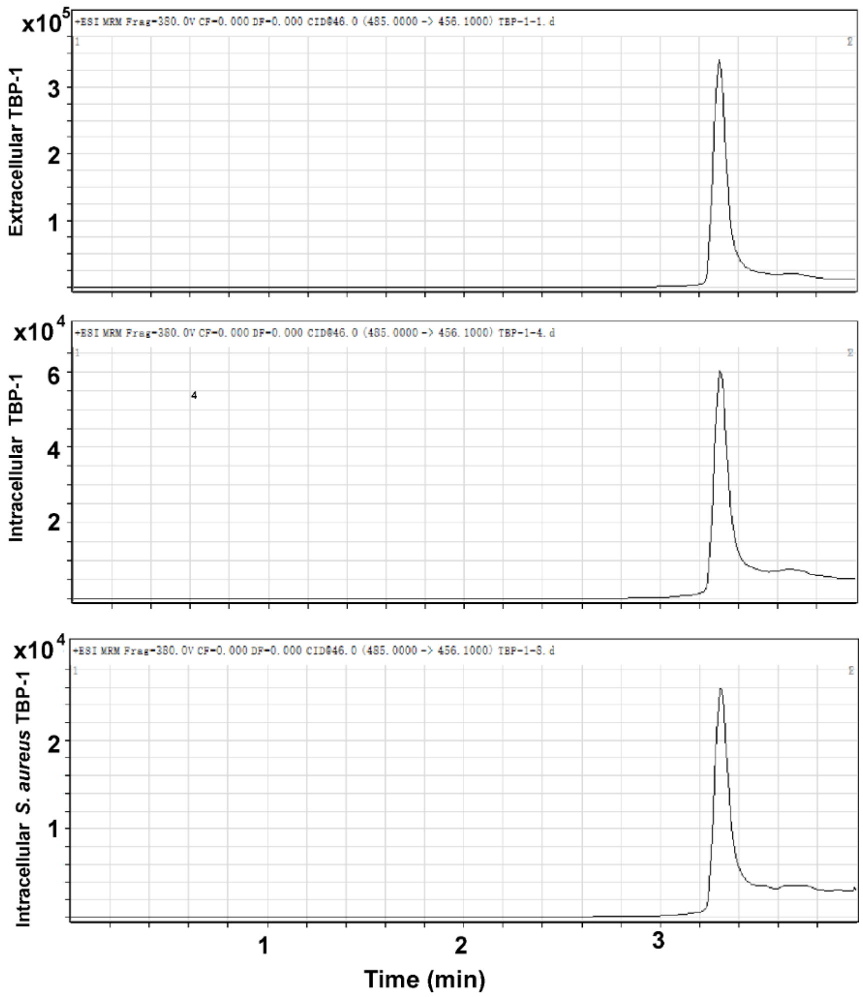

Figure S26. Quantification of the concentrations of extracellular TBP-1, internalized TBP-1, and TBP-1 in the cytosol of IEC-6 cells infected with *S.aureus* by liquid chromatography-tandem mass analysis (LC-MS/MS).

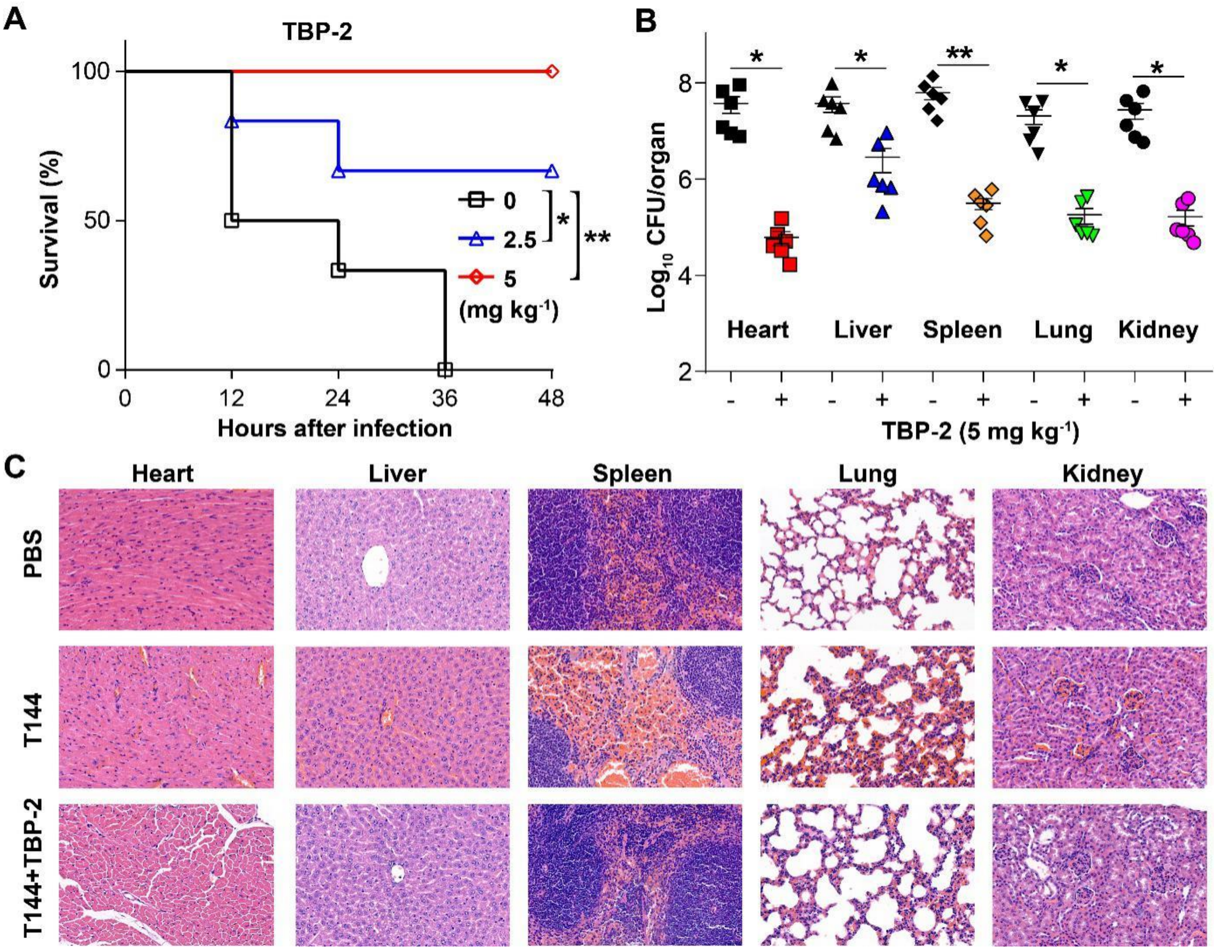

Figure S27. Efficacy of TBP-2 in the mouse peritonitis model. Survival rate (A) and bacteria survival (B) in different organs of mice after treatment with TBP-2 in septicemia protection model using MRSA T144 (n= 5, *P<0.05; **P <0.001). C) Histological staining images of heart, liver, spleen, lung and kidney tissue sections at day 2 after different treatments indicated.

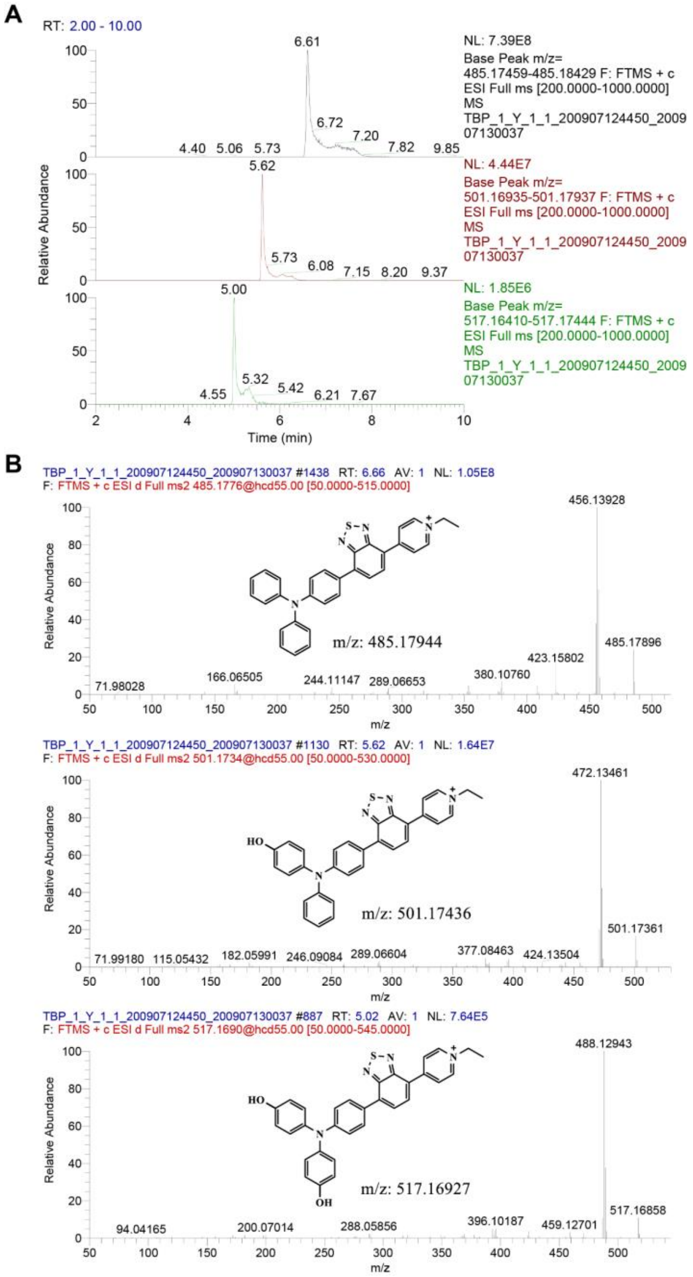

Figure S28. Metabolic Pathways of TBP-1 in in Vitro Systems. A) Extracted mass chromatography of the metabolites of TBP-1 detected in *in vitro* systems. B) MS/MS spectra of the metabolites of TBP-1 detected in *in vitro* systems of rats, and metabolic molecules of TBP-1 toxin in *in vitro* systems.
